# Reporting of Observational Studies Explicitly Aiming to Emulate Randomized Trials: A Systematic Review

**DOI:** 10.1001/jamanetworkopen.2023.36023

**Published:** 2023-09-27

**Authors:** Harrison J. Hansford, Aidan G. Cashin, Matthew D. Jones, Sonja A. Swanson, Nazrul Islam, Susan R. G. Douglas, Rodrigo R. N. Rizzo, Jack J. Devonshire, Sam A. Williams, Issa J. Dahabreh, Barbra A. Dickerman, Matthias Egger, Xabier Garcia-Albeniz, Robert M. Golub, Sara Lodi, Margarita Moreno-Betancur, Sallie-Anne Pearson, Sebastian Schneeweiss, Jonathan A. C. Sterne, Melissa K. Sharp, Elizabeth A. Stuart, Miguel A. Hernán, Hopin Lee, James H. McAuley

**Affiliations:** 1 School of Health Sciences, Faculty of Medicine and Health, UNSW Sydney, Sydney, Australia; 2 Centre for Pain IMPACT, Neuroscience Research Australia, Sydney, Australia; 3 Department of Epidemiology, University of Pittsburgh, Pittsburgh, Pennsylvania; 4 Oxford Population Health, Big Data Institute, University of Oxford, Oxford, United Kingdom; 5 Faculty of Medicine, University of Southampton, Southampton, United Kingdom; 6 CAUSALab, Harvard T.H. Chan School of Public Health, Boston, Massachusetts; 7 Department of Epidemiology, Harvard T.H. Chan School of Public Health, Boston, Massachusetts; 8 Department of Biostatistics, Harvard T.H. Chan School of Public Health, Boston, Massachusetts; 9 Institute of Social and Preventive Medicine, University of Bern, Bern, Switzerland; 10 Centre for Infectious Disease Epidemiology and Research, Faculty of Health Sciences, University of Cape Town, Cape Town, South Africa; 11 Population Health Sciences, Bristol Medical School, University of Bristol, Bristol, United Kingdom; 12 RTI Health Solutions, Barcelona, Spain; 13 Department of Medicine, Northwestern University Feinberg School of Medicine, Chicago, Illinois; 14 Department of Biostatistics, Boston University School of Public Health, Boston, Massachusetts; 15 Clinical Epidemiology & Biostatistics Unit, Murdoch Children’s Research Institute, Royal Children’s Hospital, Parkville, Victoria, Australia; 16 Department of Paediatrics, The University of Melbourne, Parkville, Victoria, Australia; 17 School of Population Health, Faculty of Medicine and Health, UNSW Sydney, New South Wales, Australia; 18 Division of Pharmacoepidemiology, Department of Medicine, Brigham & Women’s Hospital, Harvard Medical School, Boston, Massachusetts; 19 Department of Population Health Sciences, Bristol Medical School, University of Bristol, Bristol, United Kingdom; 20 NIHR Bristol Biomedical Research Centre, Bristol, United Kingdom; 21 Health Data Research UK South-West, Bristol, United Kingdom; 22 Department of Public Health and Epidemiology, RCSI University of Medicine and Health Sciences, Dublin, Ireland; 23 Department of Biostatistics, Johns Hopkins Bloomberg School of Public Health, Baltimore, Maryland; 24 University of Exeter Medical School, Exeter, United Kingdom

## Abstract

**Importance:**

Observational (nonexperimental) studies that aim to emulate a randomized trial (ie, the target trial) are increasingly informing medical and policy decision-making, but it is unclear how these studies are reported in the literature. Consistent reporting is essential for quality appraisal, evidence synthesis, and translation of evidence to policy and practice.

**Objective:**

To assess the reporting of observational studies that explicitly aimed to emulate a target trial.

**Evidence Review:**

We searched Medline, Embase, PsycINFO, and Web of Science for observational studies published between March 2012 and October 2022 that explicitly aimed to emulate a target trial of a health or medical intervention. Two reviewers double-screened and -extracted data on study characteristics, key predefined components of the target trial protocol and its emulation (eligibility criteria, treatment strategies, treatment assignment, outcome[s], follow-up, causal contrast[s], and analysis plan), and other items related to the target trial emulation.

**Findings:**

A total of 200 studies that explicitly aimed to emulate a target trial were included. These studies included 26 subfields of medicine, and 168 (84%) were published from January 2020 to October 2022. The aim to emulate a target trial was explicit in 70 study titles (35%). Forty-three studies (22%) reported use of a published reporting guideline (eg, Strengthening the Reporting of Observational Studies in Epidemiology). Eighty-five studies (43%) did not describe all key items of how the target trial was emulated and 113 (57%) did not describe the protocol of the target trial and its emulation.

**Conclusion and Relevance:**

In this systematic review of 200 studies that explicitly aimed to emulate a target trial, reporting of how the target trial was emulated was inconsistent. A reporting guideline for studies explicitly aiming to emulate a target trial may improve the reporting of the target trial protocols and other aspects of these emulation attempts.

## Key Points

**Question:**

How are studies that explicitly aim to emulate a target trial reported?

**Findings:**

In this systematic review of 200 studies that explicitly aimed to emulate a target trial, reporting was inconsistent, and studies often did not report all necessary information related to the emulation of the target trial.

**Meaning:**

Inconsistent reporting of studies that explicitly aim to emulate a target trial may impair the appraisal, synthesis, and implementation of study findings.

## Teaser

This systematic review assesses the reporting of observational studies that explicitly aimed to emulate target trials, hypothetical randomized trials that could answer causal questions of interest.

## Introduction

Analyses of observational (nonexperimental) data can be used to estimate the causal effect of interventions when randomized clinical trials are unavailable or infeasible. Bias in observational analyses may be limited by conceptualizing them as attempts to emulate target trials, ie, hypothetical randomized trials that would answer causal questions of interest.^1,2,3^ Hernán and Robins^4^ have outlined a framework for this approach, which involves first specifying the protocol of the target trial and then emulating the trial as closely as possible using observational data.^4,5^ The target trial framework may help reduce common biases in observational analyses and enhance transparency regarding design and analytic decisions. Moreover, it facilitates the interpretation of effect estimates and promotes meaningful discourse concerning potentially discrepant findings observed across studies.

Since at least the 1950s, the notion of observational analyses as attempts to approximate the goals of randomized clinical trials has underpinned many comparative studies in health, medicine, and related fields.^6,7,8,9,10^ The target trial emulation framework, introduced by Hernán and Robins^4^ in 2016, provided a template for reporting and conducting studies that aim to emulate target trials. The framework outlines items to be reported in the protocol of a target trial and its emulation, including: eligibility criteria, treatment strategies, assignment procedures, follow-up period, outcome(s), causal contrast(s), and analysis plan. Since the introduction of the framework, several articles have been published to assist researchers in conducting these studies and educating clinicians and other end users to interpret their findings.^3,5,11,12,13,14^ However, there is limited understanding of how researchers have implemented the target trial framework when reporting observational analyses with the explicit aim to emulate a target trial.

This review aimed to (1) describe how studies that explicitly aimed to emulate a target trial were reported and (2) examine whether these used published reporting guidelines. The findings of this review will be used to inform the development of a reporting guideline for studies explicitly aiming to emulate a target trial.^15^

## Methods

This review is reported according to the Preferred Reporting Items for Systematic Reviews and Meta-analyses (PRISMA) reporting guideline.^16^ The protocol was prospectively registered on the Open Science Framework (OSF).^17,18^ Protocol deviations are reported in eAppendix 1 in Supplement 1.

### Searches

We searched 4 electronic databases of published literature from March 13, 2012, to October 20, 2022, including Medline, Embase, PsycINFO, and Web of Science. Our search included terms such as emulat* trial, *target trial*, target trial emulat*, *real world data*, and *causal inference*. Our complete search strategy is provided in eAppendix 2 in Supplement 1. To supplement the search strategy, we used citationchaser^19^ to conduct forward citation tracking of 5 selected seminal papers describing the target trial emulation framework.^3,4,8,20,21^ We also included papers known to the authorship team.

### Eligibility Criteria

We included observational studies that explicitly aimed to emulate a target trial of a medical intervention; eAppendix 3 in Supplement 1 provides all terms deemed sufficient for an explicit target trial emulation. We restricted our inclusion of studies published from March 13, 2012 (10 years prior to registration of our protocol^18^ to capture recent trends in reporting) to October 20, 2022. We excluded studies that did not investigate a medical intervention; did not include human participants; were not written in English; only described the protocol of a study emulating a target trial, ie, a protocol of a planned study without results; or for which the full text was unavailable.

### Record Management and Screening

We de-duplicated all records identified through searches in Endnote version 20 and imported into Excel version 2206 (Microsoft Corp). In duplicate, reviewers (H.J.H., A.G.C., M.D.J., and S.R.G.D.) independently performed screening of identified records for eligibility at the level of title and abstract and full text. Disagreements were resolved through discussion.

### Data Extraction

Data were extracted in duplicate (H.J.H., M.D.J., S.R.G.D., J.J.D., S.A.W., R.R.N.R., A.G.C. performed this task independently) and compiled into a standardized spreadsheet piloted with 3 included studies. Disagreements were resolved by the lead author (H.J.H.) or through discussion. We did not blind reviewers to the journal article or study authors.

### Data Items

We extracted information about the (1) characteristics of the included studies, (2) key protocol components that characterize the target trial approach, and (3) further items that may be important to report in studies emulating a target trial. The complete data extraction spreadsheet and code used are available on OSF.^18^

Characteristics of the included studies were year of publication, subfield of medicine defined based upon included population, data source (prospective cohort, electronic health records, claims data, registry, randomized clinical trial, or linked data, ie, where data sources were combined), sample size (unique individuals included and analyzed, rather than simulated or duplicated persons, such as in sequential trial designs), primary outcome, and type of treatment strategy being compared. *Treatment strategy* refers to any health care intervention including treatments, preventative interventions, and no change to current treatments, remaining consistent with the language used by Hernán and Robins.^4^ If a study investigated prevention of a given outcome in healthy individuals, the subfield of medicine was designated based on the outcome investigated. Each treatment strategy included in an article (eg, ≥2) was counted separately. We classified treatment strategies defined by the authors as no treatment or usual care as no change to current treatment approach(es).

We extracted whether each study reported the eligibility criteria, treatment strategies, assignment procedures, follow-up period, outcome(s), causal contrast(s), and analysis plan of the emulation of the target trial. These items and their definitions were informed by the target trial framework from Hernán and Robins.^4^ We considered a study to have specified how the target trial was emulated if all the previously listed protocol items were reported; these items are operationalized in eAppendix 4 in the Supplement. We extracted whether the protocol of the target trial or its emulation were presented in a table or in text, with table being prioritized if reported in both table and text format. We also stated whether the protocol of the target trial and how it was emulated were reported. We extracted whether the study reported a baseline in the target trial emulation where eligibility criteria, start of follow-up, and treatment assignment were aligned.

Further details of specific protocol components that may be important to report in studies emulating a target trial were chosen based on expert knowledge and recommendations from methodological papers on the target trial emulation framework.^2,3,4^ These included:

- Treatment strategies: type of treatment strategy (eg, pharmacological, surgical; all studies are expected to include 2 or more treatment strategies), aspects of treatment strategies described (type of treatment, frequency, dose, and duration of treatment strategy).
- Analysis plan: method(s) used to emulate randomization, description and selection of potential confounding variables, statistical and causal assumptions that relate to analyses, sensitivity analyses.
- Other: study registration, rationale for the target trial emulation, reporting guideline used (referred to as a guideline hereafter). We only included a guideline if it was referenced as guiding the reporting of a study.

We deemed a study to report the assumptions underlying their analyses only when the assumption(s) were described in the text or in a cited reference. When authors reported that no residual confounding was assumed, we took this as equivalent to reporting an assumption of conditional exchangeability. We did not regard practices that may assess a causal assumption (eg, truncation of weights to satisfy the assumption of positivity) as reporting the assumption. We did not assess the appropriateness of authors’ reported assumptions. Items we extracted that were not included in commonly used guidelines are listed in eAppendix 5 in Supplement 1.

### Data Analysis

We cleaned and analyzed data in R version 4.2.0 (R Project for Statistical Computing) using tableone, openxlsx, tidyverse, and readxl packages for data management and visualization. We summarized categorical variables using counts and percentages. Continuous variables were summarized using mean and SD or median and interquartile range. Post hoc, we assessed the reporting of how the target trial was emulated stratified by whether a guideline was used.

## Results

**Figure 1.**
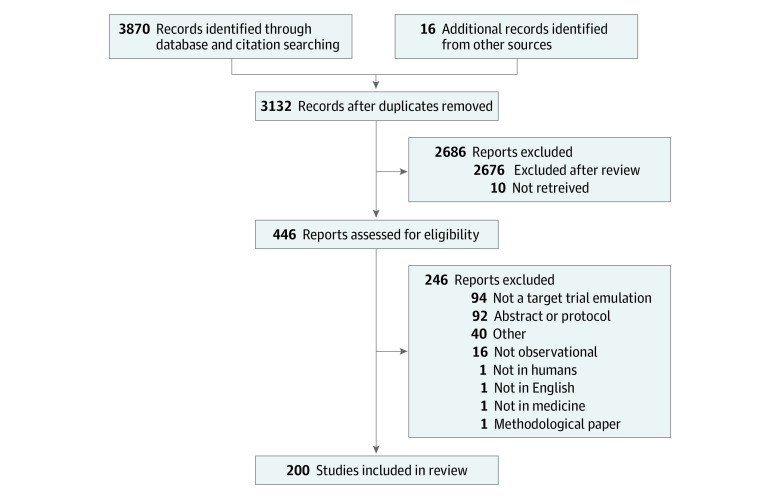
Study Flow Diagram

We retrieved 3133 unique records, of which 200 were included in the review (Figure 1).^2,22,23,24,25,26,27,28,29,30,31,32,33,34,35,36,37,38,39,40,41,42,43,44,45,46,47,48,49,50,51,52,53,54,55,56,57,58,59,60,61,62,63,64,65,66,67,68,69,70,71,72,73,74,75,76,77,78,79,80,81,82,83,84,85,86,87,88,89,90,91,92,93,94,95,96,97,98,99,100,101,102,103,104,105,106,107,108,109,110,111,112,113,114,115,116,117,118,119,120,121,122,123,124,125,126,127,128,129,130,131,132,133,134,135,136,137,138,139,140,141,142,143,144,145,146,147,148,149,150,151,152,153,154,155,156,157,158,159,160,161,162,163,164,165,166,167,168,169,170,171,172,173,174,175,176,177,178,179,180,181,182,183,184,185,186,187,188,189,190,191,192,193,194,195,196,197,198,199,200,201,202,203,204,205,206,207,208,209,210,211,212,213,214,215,216,217,218,219,220^ All reasons for excluding records after full-text review are given in eAppendix 6 in Supplement 1.

### Characteristics of Included Studies

**Figure 2.**
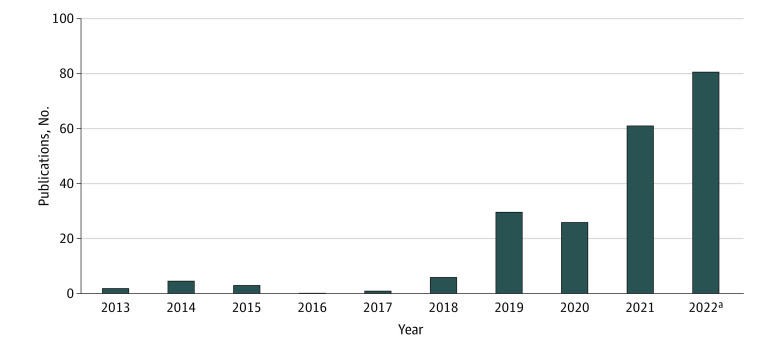
Number of Explicit Emulations of a Target Trial Included in Review Published per Year

Of the 200 included studies,^2,22,23,24,25,26,27,28,29,30,31,32,33,34,35,36,37,38,39,40,41,42,43,44,45,46,47,48,49,50,51,52,53,54,55,56,57,58,59,60,61,62,63,64,65,66,67,68,69,70,71,72,73,74,75,76,77,78,79,80,81,82,83,84,85,86,87,88,89,90,91,92,93,94,95,96,97,98,99,100,101,102,103,104,105,106,107,108,109,110,111,112,113,114,115,116,117,118,119,120,121,122,123,124,125,126,127,128,129,130,131,132,133,134,135,136,137,138,139,140,141,142,143,144,145,146,147,148,149,150,151,152,153,154,155,156,157,158,159,160,161,162,163,164,165,166,167,168,169,170,171,172,173,174,175,176,177,178,179,180,181,182,183,184,185,186,187,188,189,190,191,192,193,194,195,196,197,198,199,200,201,202,203,204,205,206,207,208,209,210,211,212,213,214,215,216,217,218,219,220^ 168 (84%) were published from January 2020 to October 2022 (Figure 2).^22,23,25,26,27,28,29,30,31,32,33,34,35,36,37,38,39,40,41,42,43,44,45,46,48,49,50,51,53,57,58,60,61,62,63,64,65,66,67,68,72,73,74,75,76,77,79,80,82,83,84,85,86,87,88,89,90,94,95,96,97,99,100,101,102,103,104,105,106,107,108,109,110,111,112,113,115,116,117,118,119,120,121,122,123,124,125,126,127,128,129,130,131,132,133,134,135,136,137,138,139,140,141,142,143,145,146,147,148,149,150,151,152,153,154,155,156,157,158,159,160,161,162,163,164,165,167,168,169,170,171,172,173,174,175,176,177,178,179,181,183,184,185,186,187,188,189,190,191,192,193,194,195,197,198,199,201,203,205,206,208,210,211,213,214,215,216,217^ The included studies spanned 26 fields of medicine, predominately infectious disease (43 [22%]; 27 [14%] on COVID-19),^26,27,30,35,36,37,38,39,47,53,54,55,56,59,66,67,68,77,82,90,94,97,99,100,108,109,110,130,136,139,140,141,145,148,157,166,186,197,205,217,218^ cardiology (30 [15%]),^22,28,31,41,60,70,71,73,85,105,106,116,117,121,122,143,149,150,151,152,161,179,184,187,193,198,206,214^ and oncology (30 [15%]).^2,32,40,44,45,46,51,76,78,81,89,91,92,93,95,103,104,129,132,138,153,168,173,174,178,182,190,192,204,211^ One hundred and thirty-one studies (66%)^2,22,23,25,31,32,34,35,36,39,41,42,43,44,45,46,47,51,52,53,58,59,61,62,63,65,67,68,70,71,72,73,74,75,76,77,80,81,85,86,89,90,92,93,94,95,96,100,101,102,103,104,105,106,108,109,111,112,116,117,119,120,121,122,123,124,125,126,127,128,129,130,131,133,134,135,136,137,141,143,144,146,147,148,149,150,151,152,153,154,155,156,157,158,160,161,162,164,167,168,170,171,173,177,178,179,180,181,183,184,185,187,188,189,193,195,199,200,201,205,207,210,212,213,216,217,219,220^ used data from electronic health records, claims databases, or linked data sources. The treatment strategies most frequently investigated were pharmacological (228 of 435 [52%]) and no change to current treatment approach(es) (usual care or noninitiation of study treatment; 82 of 435 [19%]). All extracted characteristics of included studies are displayed in Table 1.

**Table 1.** Characteristics of 200 Included Studies

Characteristic	Count, No. (%)
Domain	
Infectious diseases	43 (22)
Cardiology	30 (15)
Oncology	30 (15)
Nephrology	14 (7)
Endocrinology	11 (6)
Rheumatology	10 (5)
Internal medicine	9 (5)
Neurology	7 (4)
Psychiatry	6 (3)
Other	40 (20)
Data source	
Electronic health record data	49 (25)
Linked data^a^	46 (23)
Claims data	36 (18)
Registry	32 (16)
Prospective cohort^b^	30 (15)
Randomized clinical trial	6 (3)
Not reported	1 (1)
Sample size, median (IQR)^c^	
Sample eligible	11 253 (2157-101 078)
Sample analyzed	9799 (1995-98 718)
Primary outcome	
Death	72 (36)
Major adverse cardiovascular event^d^	19 (10)
Cancer	8 (4)
Other	101 (51)
Treatment strategies compared, No.	
2	187 (94)
3	5 (3)
4	4 (2)
5	2 (1)
6	2 (1)
10	1 (1)
Type of treatment strategy, No./total No.(%)^e^	
Pharmacological	228/435 (52)
No change to current treatment	82/435 (19)
Other^f^	61/435 (14)
Surgical	37/435 (9)
Vaccine	19/435 (4)
Medical device	8/435 (2)

^a^ Data in which 2 or more data sources are combined, eg, a registry is linked to a claims database.

^b^ Studies could only contribute to 1 data source item; if data collection for a cohort was conducted prospectively, the data source was classified only as a prospective cohort, even if data collection took place in the form of electronic health records or other data source listed.

^c^ The sample size includes the number of unique participants.

^d^ As major adverse cardiovascular event is often described heterogeneously, myocardial infarction, stroke, or major adverse cardiovascular event as defined by the authors were included; where the primary outcome was death, even if from cardiovascular events, the outcome was classified as death.

^e^ A given study may contribute 2 or more treatment strategies, which may be different, ie, 2 pharmacological treatment strategies compared with 2 no change to current treatment strategies.

^f^ The other category includes health care consultations, health care programs, organ transplants, and other interventions that would not fall under the other categories listed.

### Characteristics of Target Trials and How They Were Emulated

One-hundred and fifteen studies (58%)^2,22,23,25,26,30,31,32,34,35,42,45,46,47,48,49,50,51,54,55,56,57,59,61,64,65,66,69,70,71,72,74,75,76,77,78,85,87,88,89,90,91,93,95,96,98,100,101,102,103,104,106,116,127,128,130,132,133,134,135,137,138,139,141,142,143,148,149,151,152,154,156,157,158,160,161,162,167,168,172,174,176,177,178,179,180,182,183,184,187,188,189,190,191,192,195,196,198,199,200,201,203,204,205,206,208,209,210,211,213,214,215,216,219,220^ completely reported how the target trial protocol was emulated. Eighty-seven studies (44%)^2,24,25,28,30,32,34,42,46,51,52,55,56,57,59,61,62,64,65,66,70,71,72,76,77,78,79,82,85,87,88,89,91,92,93,94,98,100,103,104,107,113,115,116,117,119,120,122,125,126,130,133,134,135,142,143,147,149,150,152,158,162,165,168,170,172,176,179,185,187,188,190,191,192,195,202,203,209,212,213,214,216,219,220^ provided both the protocol of the target trial and described how it was emulated (Table 2). The following items of the emulation were frequently reported: eligibility criteria (193 [97%]), treatment strategies (191 [96%]), assignment procedures (173 [87%]), primary outcome (196 [98%]), the follow-up period (186 [93%]), a causal contrast (146 [73%], and an analysis plan (194 [97%]) (Table 2).

**Table 2.** Characteristics of Target Trials and How They Were Emulated

Characteristic	Count, No. (%)
How the protocol of the emulated target trial was reported	
Not fully described	85 (42)
Only in text	59 (30)
Table	56 (28)
Both target trial protocol and its emulation described explicitly as such	87 (44)
Description of how the target trial was emulated^a^	
Eligibility criteria	193 (97)
Treatment strategies	191 (96)
Assignment procedures	173 (87)
Outcome(s)	196 (98)
Follow-up	186 (93)
Causal contrast(s)	146 (73)
Analysis plan	194 (97)
Specification of time zero (ie, baseline)	165 (83)

^a^ Operational definitions of target trial protocol items are described in eAppendix 4 in Supplement 1.

### Reporting of Further Items That Relate to the Target Trial Emulation

Seventy studies (35%)^23,24,25,29,30,32,34,37,43,48,50,51,55,56,57,59,61,62,65,66,70,71,74,75,76,82,86,96,98,100,103,107,109,110,113,116,126,129,130,134,135,139,143,145,146,147,149,153,154,158,159,163,165,167,168,171,172,174,175,177,179,189,199,203,206,207,208,213,216,218^ reported in the title that the study aimed to emulate a target trial; 180 (90%)^2,22,23,24,25,26,27,30,31,32,33,34,35,36,37,38,39,40,41,42,43,44,45,46,48,49,50,51,52,53,54,55,56,57,58,59,60,61,62,63,64,65,66,67,68,69,70,71,72,73,74,75,76,77,78,79,80,81,82,83,84,86,87,88,89,91,92,93,94,95,96,97,99,100,101,102,103,104,106,107,109,110,111,112,113,115,116,117,118,119,121,122,123,124,125,126,127,128,130,131,132,133,134,135,136,137,138,139,140,141,142,143,144,146,147,148,149,150,151,152,153,154,156,157,158,160,161,162,164,165,166,167,168,169,170,171,173,174,176,177,178,179,180,181,182,183,184,185,186,187,188,189,190,191,192,193,194,195,197,198,199,200,201,202,203,204,205,206,207,208,209,210,211,212,213,214,216,217,218,219,220^ did so in the Methods section. Twenty studies (10%) ^24,25,34,35,43,52,68,80,84,86,117,131,133,147,156,161,169,171,192,216^ reported the study was prospectively registered, 16 of these 20 (80%)^24,25,34,43,52,68,80,86,117,131,133,156,161,169,171,216^ also provided information on how to access the registration. One hundred and twenty-six studies (63%)^2,22,23,24,25,26,27,28,29,30,34,35,37,39,40,44,45,46,48,49,50,52,54,57,59,60,62,64,65,66,67,68,69,70,71,72,73,74,75,76,78,79,82,83,86,87,88,89,91,92,93,99,102,103,104,108,109,111,112,113,114,115,117,120,121,122,123,124,125,126,127,130,133,134,135,138,139,141,142,145,146,147,149,150,151,152,153,155,156,158,159,161,162,164,168,169,171,172,173,175,176,179,180,182,184,185,187,190,191,192,194,195,197,198,199,200,201,205,208,209,210,211,213,215,216,217^ specified whether a randomized clinical trial could be feasibly conducted; 61 (31%)^28,29,34,39,45,48,49,54,57,62,66,67,68,69,73,75,79,83,86,87,88,91,99,102,103,104,108,109,112,115,117,121,122,123,125,126,138,139,146,152,153,156,158,162,171,184,185,187,192,198,199,205,210,211,213,215,216^ stated that the randomized clinical trial was possible. Of the studies that stated a randomized clinical trial was possible, uncertainty in the generalizability of available trial findings was the most common reason for the target trial emulation (22 of 61 [36%]).^28,45,52,57,68,85,86,103,114,115,117,122,123,152,158,184,187,192,198,210,215^ Forty-three studies (22%)^2,27,39,45,48,53,61,62,67,75,80,87,99,101,102,104,107,111,112,114,117,118,122,123,128,130,135,144,151,152,155,158,159,160,161,168,183,189,194,195,208,210,215^ reported using a guideline, most commonly (29 of 43 [67%]) the Strengthening the Reporting of Observational Studies in Epidemiology (STROBE) guideline.^221,222^ There were no qualitative differences between the reporting of the target trial emulation when studies were stratified by guideline use (eAppendix 7 in Supplement 1).

Most studies (187 [94%])^2,22,23,24,25,26,27,28,29,31,32,33,34,35,36,37,38,39,40,42,43,44,45,46,47,48,49,50,51,53,54,55,56,57,58,59,60,61,63,64,65,66,67,68,69,70,71,72,73,74,75,76,77,78,79,81,82,83,87,89,90,91,92,93,94,95,96,97,98,99,100,101,102,103,104,105,106,107,108,109,110,111,112,113,114,115,116,117,118,119,120,121,122,123,124,125,126,128,129,130,131,132,133,134,135,136,137,138,139,140,141,142,143,144,145,146,147,148,149,150,151,152,153,154,155,156,157,158,159,160,161,162,163,164,165,166,167,168,169,170,171,172,173,174,176,177,178,179,180,181,182,183,184,186,187,188,189,190,191,192,194,195,196,197,198,199,200,201,202,203,204,205,206,207,208,209,210,211,212,213,214,215,216,217,218,219,220^ reported the set of variables that authors had decided to adjust for (eg, because they were potential confounders) in analyses, and 77 (39%)^22,24,25,26,28,35,41,44,47,48,49,50,51,52,58,61,68,69,70,77,80,81,84,87,95,99,100,104,105,107,110,111,114,124,126,130,135,138,140,141,147,148,149,151,155,156,158,159,160,161,162,165,166,168,170,171,173,175,182,183,185,186,197,199,205,208,212,214,216,217,218^ reported how these variables were selected. One hundred and thirty-one studies (66%)^2,25,26,28,31,34,36,41,42,43,44,46,47,48,49,52,54,56,57,58,59,61,64,65,66,68,69,70,72,73,75,76,77,78,80,82,83,84,85,86,87,88,90,92,93,94,95,99,100,101,105,106,107,109,112,113,114,115,116,117,119,120,123,125,126,127,128,129,130,131,133,134,135,136,137,138,141,142,143,144,146,152,153,154,157,158,160,161,162,163,164,165,166,168,169,170,171,172,173,174,177,179,180,181,187,188,189,192,194,195,196,198,199,200,201,204,205,206,207,208,210,211,212,213,214,215,216,217,218,219,220^ reported conducting a sensitivity analysis for statistical or causal assumptions; the most frequent (42 of 131 [32%]) was the use of a different approach to confounding adjustment (eg, using weighting rather than outcome regression). One hundred and fifty-eight studies (79%)^2,22,25,26,27,28,29,30,32,36,38,39,40,41,44,45,46,47,48,49,50,51,52,54,55,56,57,58,59,61,63,64,65,66,67,68,69,70,71,72,75,76,77,78,79,80,81,82,83,84,85,87,88,89,90,93,94,97,99,100,101,103,105,106,107,108,109,111,112,113,114,116,117,118,119,120,121,122,123,126,127,128,129,130,132,133,134,135,136,137,138,139,140,141,142,143,144,145,146,147,149,150,152,153,155,156,158,159,160,161,162,163,165,166,167,168,169,170,171,172,173,174,175,176,177,180,181,182,183,186,187,188,189,190,191,192,194,195,197,198,199,200,201,202,203,204,205,206,207,208,210,211,212,213,214,215,216,219^ reported that causal interpretation rests on the assumption that the comparison groups were comparable (ie, exchangeable) given the variables included in the analysis (Table 3). Twenty-six studies (13%)^29,42,48,49,56,70,71,82,85,100,119,129,135,153,162,163,165,170,172,175,187,194,200,203,208,213^ reported reliance on more than 1 causal assumption.

**Table 3.** Reporting of Further Items That Relate to the Target Trial Emulation

Item	Count, No. (%)
Where aim to emulate a target trial was described^a^	
Title	70 (35)
Abstract	148 (74)
Introduction	119 (60)
Methods	180 (90)
Results	55 (28)
Discussion	142 (71)
Study prospectively registered	20 (10)
Reason given why a randomized clinical trial could not be conducted	
Not reported	74 (37)
NA, trial possible	61 (31)
Unethical	16 (8)
Long-term follow-up	7 (4)
Rare outcomes	7 (4)
Too costly	5 (3)
Not timely	5 (3)
Other	25 (13)
When randomized clinical trial was reported as being possible, primary reason given for emulating a target trial, No./total No. (%)	
Generalizability of available trial findings	22/61 (36)
Replicate published trial	14/61 (23)
Trial ongoing	8/61 (13)
Comparative effectiveness not previously investigated	5/61 (8)
Previous conflicting results reported	3/61 (5)
Other	26/61 (43)
Data source cited	125 (63)
Reporting guideline reported	43 (22)
Reporting guideline used, No./total No. (%)	
STROBE	29/43 (67)
ISPOR Good Research Practices for Comparative Effectiveness Research^b^	5/43 (12)
RECORD	4/43 (9)
Nature Research Reporting Summary	3/43 (7)
RECORD-PE	2/43 (5)
TRIPOD	1/43 (2)
Aspects of treatment strategies described^a^	
Type	417 (96)
Dose	83 (19)
Duration	57 (13)
Frequency	54 (12)
None	18 (4)
Other	13 (3)
Variables adjusted in analyses listed	187 (94)
Potential unmeasured confounders listed	73 (37)
Method for selection of variables adjusted for described	77 (39)
Analytic and causal assumptions stated^a^	
Exchangeability given selected confounders	158 (79)
Positivity	27 (14)
Consistency	13 (7)
Statistical assumptions	24 (12)
Other	4 (2)
None	35 (18)
Sensitivity analyses attempting to assess robustness to analytic or causal assumption(s) violations given	131 (66)
Sensitivity analyses as reported by authors, No./total No. (%)	
Different approach to confounding adjustment	42/131 (32)
Negative control	23/131 (18)
Additional adjustment for confounding	19/131 (15)
E-value	15/131 (11)
Different censoring procedure	10/131 (8)
Different approach to handling missing data	4/131 (3)
Other	24/131 (18)
Table describing baseline characteristics of groups presented	171 (86)

Abbreviations: ISPOR, International Society of Pharmacoeconomics and Outcomes Research; NA, not applicable; RECORD, Reporting of Studies Conducted Using Observational Routinely Collected Health Data; RECORD-PE, Reporting of studies Conducted Using Observational Routinely Collected Health Data–Statement for Pharmacoepidemiology; STROBE, Strengthening the Reporting of Observational Studies in Epidemiology; TRIPOD, Transparent Reporting of a Multivariable Prediction Model for Individual Prognosis or Diagnosis.

^a^ Total exceeds 100% as multiple characteristics could be included in a single study.

^b^ The ISPOR Good Research Practices for Comparative Effectiveness Research are guidelines for the conduct of comparative effectiveness studies, not a reporting guideline, however, were commonly cited as being used for reporting, therefore have been included.

## Discussion

This systematic review summarized items reported in observational studies that explicitly aimed to emulate a target trial. We included 200 studies^2,22,23,24,25,26,27,28,29,30,31,32,33,34,35,36,37,38,39,40,41,42,43,44,45,46,47,48,49,50,51,52,53,54,55,56,57,58,59,60,61,62,63,64,65,66,67,68,69,70,71,72,73,74,75,76,77,78,79,80,81,82,83,84,85,86,87,88,89,90,91,92,93,94,95,96,97,98,99,100,101,102,103,104,105,106,107,108,109,110,111,112,113,114,115,116,117,118,119,120,121,122,123,124,125,126,127,128,129,130,131,132,133,134,135,136,137,138,139,140,141,142,143,144,145,146,147,148,149,150,151,152,153,154,155,156,157,158,159,160,161,162,163,164,165,166,167,168,169,170,171,172,173,174,175,176,177,178,179,180,181,182,183,184,185,186,187,188,189,190,191,192,193,194,195,196,197,198,199,200,201,202,203,204,205,206,207,208,209,210,211,212,213,214,215,216,217,218,219,220^ published from 2013 to 2022, the majority of which (168 [84%]) were published between January 2020 and October 2022. The studies spanned 26 fields of medicine and mostly used sources of data that were routinely collected, such as electronic health records, health insurance claims data, or these data linked with other data sources. While the publication of studies explicitly aiming to emulate a target trial is increasing, only 58% of included studies completely reported how the target trial protocol was emulated.

Our finding that studies aiming to emulate a target trial inconsistently reported the emulation of the target trial is similar with results of previous systematic reviews of observational studies that did not explicitly aim to emulate a target trial.^223^ Nguyen et al^223^ systematically reviewed the risk of bias in observational studies investigating the effectiveness of interventions using the ROBINS-I tool,^224^ a risk of bias tool informed by the target trial framework. The authors found that only 3% of these observational studies (2 of 77) completely specified all items of the protocol of the (implicit or explicit) target trial. A much larger proportion of our sample of studies that explicitly aimed to emulate a target trial reported how the target trial was emulated; however, many were still incompletely reported. It appears the guidance from Hernán and Robins^4^ and previous work^225,226,227,228^ has been used inconsistently or perhaps misinterpreted, leaving key elements of the target trial and its emulation unreported.^3^

Our review shows there has been an increase in the publication of studies that explicitly aim to emulate a target trial. This trend could be attributed to the growing influence of such studies in shaping policy and regulatory decisions.^229,230,231,232^ For example, in mid-2022, the UK National Institute of Health and Care Excellence released “Real-World Evidence Framework,”^229^ which emphasizes the importance of using the framework of a target trial when estimating treatment effects for regulatory decision-making using observational data.^229^ Considering the emerging role of studies explicitly emulating a target trial within the health care decision-making framework, it is critical these studies are consistently and transparently reported. Once a target trial is emulated, unmeasured confounding may be a primary concern with observational analyses informing decision-making.^229,233^ We found that only 73 studies (37%) reported potential unmeasured confounders. It is unlikely all confounders would be measured in a given analysis, therefore the robustness of findings from a target trial emulation may be better assessed if potentially important unmeasured confounders are reported.

Guidelines have been developed to address inconsistent reporting,^234^ and if actively implemented, can improve reporting consistency and completeness.^235,236,237^ None of the included studies identified specific reporting guidance for studies that aimed to explicitly emulate a target trial, and the authors are not aware of any guidance for studies emulating a target trial published or under development,^238^ suggesting no formal guidance has been published. Twenty-two percent of studies cited general (eg, STROBE)^221,222^ and potentially inappropriate guidelines (eg, Transparent Reporting of a Multivariable Prediction Model for Individual Prognosis or Diagnosis).^239^ The use of guidelines was comparatively lower than seen in similar reviews of other types of observational studies, in which observed rates of guideline use ranged from 46% (67 of 147)^240^ to 47% (68 of 88).^241^ The lower use of guidelines observed in our review may reflect authors’ uncertainty on the most appropriate guideline when reporting a study that used the target trial framework.

### Implications

Despite the growing number of studies using the target trial framework, reporting was inconsistent. Consistent and transparent reports are important for these studies given their emerging role in decision-making. For example, critical appraisal^224^ of the quality and robustness of findings from a study emulating a target trial is impaired when such analyses are poorly reported, leaving readers unable to understand the quality and conduct of the emulation. Similarly, findings of studies emulating a target trial are frequently compared with those of randomized clinical trials.^2,86,242,243,244,245^ Differences in effect estimates between target trial emulations and randomized clinical trials may arise due to various factors.^246^ Transparent reporting of the target trial protocol and how it was emulated may aid in understanding these differences and optimize the usefulness of these studies for decision-making.

No established, consensus-based^15^ guidelines are available to support authors reporting studies emulating a target trial. Commonly used guidelines (eg, STROBE)^221,222^ do not include items that relate to the protocol of the target trial^4^ or key items of the target trial emulation (causal contrast and items that relate to defining time-zero). Reporting of these items was not improved when authors followed guidelines such as STROBE (eAppendix 7 in Supplement 1). A new guideline for studies that explicitly aim to emulate a target trial is needed to provide detailed recommendations for the minimum set of items to be reported for these studies. Improved reporting of studies emulating a target trial may facilitate peer review by helping to ensure publications are complete, accurate, transparent, and reproducible. Improved reporting could also facilitate scientific discourse, support decision-making, reduce research waste, and ultimately improve health care.^247,248^

### Strengths and Limitations

We used a sensitive search strategy to ensure all relevant studies were captured and followed recommended systematic review methods,^249^ including screening studies and extracting data in duplicate. We prospectively registered this systematic review^18^ and reported the findings in line with the PRISMA 2020 reporting guideline.^16^

This study has several limitations. First, we only included studies that explicitly stated that they aimed to emulate a target trial; therefore, our findings may present a more positive view of reporting practices compared with all observational analyses comparing interventions.^250,251^ Using the target trial framework is neither necessary nor sufficient for obtaining valid causal effect estimates from observational analyses; however, the framework may guide the implementation of sound principles of causal inference and study design. Second, we prespecified the reporting items to be extracted based on published recommendations for the specification of the target trial protocol and its emulation.^4^ Therefore, our ratings for these items may be skewed toward a particular way of reporting studies explicitly emulating a target trial. Third, we did not assess the appropriateness of the methods of included studies, only their reporting.

## Conclusions

In this systematic review, reporting of studies that explicitly emulate a target trial was inconsistent, with several opportunities to improve the reporting of key items. A guideline expanding on the current recommendations may facilitate consistent and transparent reporting, improving the appraisal, synthesis, and implementation of study findings in clinical practice and health policy.

## Notes

Supplement 1. **Appendix 1.** Methodological Differences to the Protocol **Appendix 2.** Complete Search Strategies for All Databases **Appendix 3.** List of Phrases Deemed to Indicate Explicit Emulation of a Target Trial **Appendix 4.** Operational Definitions for Items of the Emulation of the Target Trial **Appendix 5.** Items Extracted That Are Not Included in Commonly Used Reporting Guidelines (STROBE, RECORD, ISPOR) **Appendix 6.** Reasons for Study Exclusion at Level of Full Text **Appendix 7.** Subgroup Analysis of Reporting of Target Trial Protocol and How It Was Emulated, by Reporting Guideline Use

Supplement 2.
Data Sharing Statement
