# Supplementary material for: Reporting of Observational Studies Explicitly Aiming to Emulate Randomized Trials: A Systematic Review
Source: JAMA Netw Open. 2023 Sep 27;6(9):e2336023. doi: 10.1001/jamanetworkopen.2023.36023 (PMC10534275; doi:10.1001/jamanetworkopen.2023.36023)
Supplement: Supplement 1. — Appendix 1. Methodological Differences to the Protocol Appendix 2. Complete Search Strategies for All Databases Appendix 3. List of Phrases Deemed to Indicate Explicit Emulation of a Target Trial Appendix 4. Operational Definitions for Items of the Emulation of the Target Trial Appendix 5. Items Extracted That Are Not Included in Commonly Used Reporting Guidelines (STROBE, RECORD, ISPOR) Appendix 6. Reasons for Study Exclusion at Level of Full Text Appendix 7. Subgroup Analysis of Reporting of Target Trial Protocol and How It Was Emulated, by Reporting Guideline Use [file jamanetwopen-e2336023-s001.pdf]

Supplemental Online Content

Hansford HJ, Cashin AG, Jones MD, et al. Reporting of observational studies explicitly emulating randomized trials: a systematic review. *JAMA Netw Open.* 2023;6(9):e2336023. doi:10.1001/jamanetworkopen.2023.36023

Supplement 1. Appendix 1. Methodological Differences to the Protocol

Appendix 2. Complete Search Strategies for All Databases

Appendix 3. List of Phrases Deemed to Indicate Explicit Emulation of a Target Trial

Appendix 4. Operational Definitions for Items of the Emulation of the Target Trial

Appendix 5. Items Extracted That Are Not Included in Commonly Used Reporting Guidelines (STROBE, RECORD, ISPOR)

Appendix 6. Reasons for Study Exclusion at Level of Full Text

Appendix 7. Subgroup Analysis of Reporting of Target Trial Protocol and How It Was Emulated, by Reporting Guideline Use

This supplemental material has been provided by the authors to give readers additional information about their work.

© 2023 Hansford HJ et al. *JAMA Network Open.*

Appendix 1. Methodological Differences to the Protocol

We initially set out to identify ‘implicit’ emulations of a target trial as well as studies which explicitly state the study as an attempt to emulate a target trial. However, there are many ways by which a study could emulate a target trial without doing so explicitly. Therefore, to contain the scope of this study, we made a post-hoc decision to exclude studies from our analysis which we initially identified as implicitly emulating a target trial. The studies excluded at this stage are listed in Appendix 5.

© 2023 Hansford HJ et al. *JAMA Network Open.*

Appendix 2. Complete Search Strategies for All Databases

Medline

1 (emulat* adj5 trial?).mp. 177

2 (target adj (trial? or experiment?)).mp. 668

3. (observational adj (stud* or research or data)).mp. 217117

4. ((real world or rwd) adj2 (stud* or research or data)).mp. 11627

5. (routine* adj2 data).mp. 8107

6. (comparative effectiveness adj2 (stud* or research or data)).mp. 5817

7. (emulat* or propensity score? or (causal adj2 (inference? or analys?s or effect*))).mp. 51359

8. 3 or 4 or 5 or 6 or 7 284549

9. 2 and 8 89

10. (target adj (trial? or experiment?)).ti. 95

11. 1 or 9 or 10 254

Filtered for time (2012-2022) manually after search

Embase

1. (emulat* adj5 trial?).mp.

2. (target adj (trial? or experiment?)).mp.

3. (observational adj (stud* or research or data)).mp.

4. ((real world or rwd) adj2 (stud* or research or data)).mp.

5. (routine* adj2 data).mp.

6. (comparative effectiveness adj2 (stud* or research or data)).mp.

7. (emulat* or propensity score? or (causal adj2 (inference? or analys?s or effect*))).mp.

8. 3 or 4 or 5 or 6 or 7

9. 2 and 8

10. (target adj (trial? or experiment?)).ti.

11. 1 or 9 or 10

psycINFO

noft(target trial emulat*) OR ((noft(real world data) OR (noft(emulat* trial)) OR noft(observational) OR noft(routine* data)) AND noft(comparative effective*) AND noft(causal infer*))

© 2023 Hansford HJ et al. *JAMA Network Open.*

Web of Science

(TI=(emulat* trial)) OR (TI=(real world data) OR TI=(routine* data) OR TI=(comparative effectiveness study comparative effectiveness research or comparative effectiveness data) OR (TI=(emulat* or propensity score?) AND TI=(causal inference or causal analysis or causal effect*))) AND ALL=(target trial or emulat* or target trial emulation)

note: ‘?’ is used as a wildcard symbol to capture different spellings

© 2023 Hansford HJ et al. *JAMA Network Open.*

Appendix 3. List of Phrases Deemed to Indicate Explicit Emulation of a Target Trial

Target trial emulation, emulate* *near* target trial, emulat* trial, emulat* *near* randomi?ed trial, trial emulation, randomi?ed trial emulation, hypothetical trial emulation, trial replication.

note: ‘?’ is used as a wildcard symbol to capture different spellings

© 2023 Hansford HJ et al. *JAMA Network Open.*

Appendix 4. Operational Definitions for Items of the Emulation of the Target Trial

Protocol element	Requirements to be deemed reported
Eligibility criteria	Stated all characteristics that define inclusion in the target trial
Treatment strategies	Stated the treatment strategies under comparison
Treatment assignment	Stated that randomization was assumed conditional on baseline covariates
Outcome(s)	Stated the outcome(s) of interest
Follow-up	Described the observation period of participants, including when follow up starts and ends
Causal contrast(s) of interest	Stated the estimand(s) of interest: e.g., intention-to-treat effect, per-protocol effect
Analysis plan	Described the statistical analyses, including methods by which covariates were adjusted to emulate randomization, e.g., matching, standardization, inverse-probability weighting

© 2023 Hansford HJ et al. *JAMA Network Open.*

Appendix 5.: Items Extracted That Are Not Included in Commonly Used Reporting Guidelines (STROBE, RECORD, ISPOR)

- Does it state whether the study was preregistered?
- Is a reason given that an RCT was not possible?
- Does the study describe itself as a target trial emulation?
- In regard to the definition of treatment strategy, which of the following do the study report: duration, type, frequency, dose, other, none)
- In what form does the study present the target trial / emulated protocol?
- **Assignment procedures**: Stated that treatment strategies were assigned based upon the assumption that randomization is being adequately emulated, or that baseline variables were adjusted for, given the baseline covariates are reported.
- **Time zero**: Does the study explicitly report how time zero was defined?
- **Causal contrast(s):** Does the study explicitly report the causal contrasts / estimands of interest?
- Does the study report if there are any potential unmeasured confounders?
- Does the study report how confounders were selected?
- Which casual assumptions are reported in the paper?
- Does the study describe similarities / differences between the ideal hypothetical randomized trial and emulation?
- Is a table comparing characteristics of participants between the two (or more) treatment groups presented?

© 2023 Hansford HJ et al. *JAMA Network Open.*

Appendix 6. Reasons for Study Exclusion at Level of Full Text

Not in English language (1)

1. Pigeot I, Kollhorst B, Didelez V. [Secondary Data for Pharmacoepidemiological Research - Making the Best of It!]. *Gesundheitswesen*. Nov 2021;83(S 02):S69-s76. Nutzung von Sekundärdaten für die pharmakoepidemiologische Forschung – machen wir das Beste draus! doi:10.1055/a-1633-3827

Not in humans (1)

2. Van Cuong NK, B. T.: Phu, D. H.: Van, N. T. B.: Hien, V. B.: Thwaites, G.: Carrique-Mas, J.: Choisy, M. Effects of prophylactic and therapeutic antimicrobial uses in small-scale chicken flocks. *Zoonoses and Public Health*. August 2021;doi:http://dx.doi.org/10.1111/zph.12839

Not in field of medicine (1)

3. Vagni GB, Richard. Earnings and Income Penalties for Motherhood: Estimates for British Women Using the Individual Synthetic Control Method. *European Sociological Review*. 2021;doi:10.1093/esr/jcab014

Not an observational study (16)

4. Atkinson AK, Michael G.: Clayton, Tim: Carpenter, James R. Reference-based sensitivity analysis for time-to-event data. *Pharmaceutical statistics*. 2019;doi:10.1002/pst.1954
5. Dehaene I, Roelens K, Smets K, Decruyenaere J. Relevance of the antenatal corticosteroids-to-delivery interval in the prevention of neonatal respiratory distress syndrome through the eyes of causal inference: a review and target trial. *Archives of Gynecology and Obstetrics*. April 2022;305(4):885-892. doi:https://dx.doi.org/10.1007/s00404-021-06213-5
6. Hsu GGM, Eric: Scheuer, Nicolas: Ramagopalan, Sreeram V. Keeping it real: implications of real-world treatment outcomes for first-line immunotherapy in metastatic non-small-cell lung cancer. *Journal*. 2021;doi:http://dx.doi.org/10.2217/imt-2021-0237
7. Jang H, Lee YB, Moon H, et al. Aspirin use and risk of hepatocellular carcinoma in patients with chronic hepatitis B with or without cirrhosis. *Hepatology (Baltimore, Md)*. 2022;76(2):492-501. doi:10.1002/hep.32380
8. Mack CD, Pavesio A, Kelly K, et al. Breaking barriers: studying fracture healing in the BONES program. *Journal of Orthopaedic Trauma*. 2021;35:S22-S27.
9. Maguen S, Madden E, Holder N, et al. Effectiveness and comparative effectiveness of evidence-based psychotherapies for posttraumatic stress disorder in clinical practice. *Psychol Med*. May 18 2021:1-10. doi:10.1017/s0033291721001628
10. Persson E, Waernbaum I, Lind T. Estimating marginal causal effects in a secondary analysis of case-control data. *Statistics in Medicine*. 2017;36(15):2404-2419. doi:https://doi.org/10.1002/sim.7277

© 2023 Hansford HJ et al. *JAMA Network Open.*

11. Sachs MCS, A.: Gabriel, E. E. Aim for Clinical Utility, Not Just Predictive Accuracy. *Epidemiology*. 1-May 2020;doi:http://dx.doi.org/10.1097/EDE.0000000000001173

12. Simmonds KP, Burke J, Kozlowski AJ, Andary M, Luo Z, Reeves MJ. Rationale for a Clinical Trial That Compares Acute Stroke Rehabilitation at Inpatient Rehabilitation Facilities to Skilled Nursing Facilities: Challenges and Opportunities. *Archives of Physical Medicine and Rehabilitation*. 2021/09/01/ 2021;doi:https://doi.org/10.1016/j.apmr.2021.08.004

13. Smith MJ, Mansournia MA, Maringe C, et al. Introduction to computational causal inference using reproducible Stata, R, and Python code: A tutorial. *Statistics in Medicine*. 2022;41(2):407-432. doi:https://doi.org/10.1002/sim.9234

14. Spera EG, Giovanni: Allegra, Dario: Stanco, Filippo: Maugeri, Andrea: Quattrocchi, Annalisa: Barchitta, Martina: Agodi, Antonella. CBMS - Randomized G-Computation Models in Healthcare Systems. *2018 IEEE 31st International Symposium on Computer-Based Medical Systems (CBMS)*. 2018;doi:10.1109/cbms.2018.00021

15. Stensrud MJ, Young JG, Didelez V, Robins JM, Hernán MA. Separable Effects for Causal Inference in the Presence of Competing Events. *Journal of the American Statistical Association*. 2022/01/02 2020;117(537):175-183. doi:10.1080/01621459.2020.1765783

16. Stensrud MJS, Louisa H. Identification of vaccine effects when exposure status is unknown. *arXiv: Methodology*. 2021;doi:NA

17. Tobias DKL, M. What would the trial be? Emulating randomized dietary intervention trials to estimate causal effects with observational data. *American Journal of Clinical Nutrition*. 1-Aug 2021;doi:http://dx.doi.org/10.1093/ajcn/nqab169

18. Usman MSP, B.: Butler, J. Target trial emulations: bridging the gap between clinical trial and real-world data. *European Journal of Heart Failure*. October 2021;doi:http://dx.doi.org/10.1002/ejhf.2331

19. Yusuf E, Rosendaal FR. Valid Evidence for Diagnosis and Treatment of Infections in the Intensive Care Unit: Beyond Randomized Control Trial Study Design (Trial Emulation and Machine Learning). Editorial. *Journal of Clinical Medicine*. July-1 2022;11(13) (no pagination)3600. doi:https://dx.doi.org/10.3390/jcm11133600

Not a target trial emulation (92)

20. Bakker LJ, Goossens LM, O'Kane MJ, Uyl-de Groot CA, Redekop WK. Analysing electronic health records: The benefits of target trial emulation. *Health Policy and Technology*. 2021;10(3):100545.

21. Bakker LJG, L. M. A.: O'Kane, M. J.: Uyl-de Groot, C. A.: Redekop, W. K. Analysing electronic health records: The benefits of target trial emulation. *Health Policy and Technology*. September 2021;doi:http://dx.doi.org/10.1016/j.hlpt.2021.100545

22. Beaudoin FLG, Roee: Merchant, Roland C.: Clark, Melissa A.: Swor, Robert A.: Jones, Jeffrey S.: Lee, David C.: Peak, David A.: Domeier, Robert M.: Rathlev, Niels K.: McLean, Samuel A. Persistent pain after motor vehicle collision: comparative effectiveness of opioids vs nonsteroidal antiinflammatory drugs prescribed from the emergency department-a propensity matched analysis. *Pain*. February 2017: 2022-01-27 2017;doi:http://dx.doi.org/10.1097/j.pain.0000000000000756

© 2023 Hansford HJ et al. *JAMA Network Open.*

23. Bijlsma MJW, Ben: Tarkiainen, Lasse: Myrskylä, Mikko: Martikainen, Pekka. The Impact of Unemployment on Antidepressant Purchasing: Adjusting for Unobserved Time-constant Confounding in the g-Formula. *Epidemiology (Cambridge, Mass)*. 2019;doi:10.1097/ede.0000000000000985
24. Brennan BP, Schnabel J, Pope HG, Hudson JI. Antidepressant use and risk of intubation or death in hospitalized patients with COVID-19: A retrospective cohort study of clinical effectiveness. *Frontiers in Psychiatry*. 2022;13(NA):NA-NA. doi:10.3389/fpsyt.2022.951065
25. Brenner SKOG, B. P.: Krajewski, M. L.: Charytan, D. M.: Chaudhry, S.: Mirza, S. H.: Peev, V.: Anderson, M.: Bansal, A.: Hayek, S. S.: Srivastava, A.: Mathews, K. S.: Johns, T. S.: Leonberg-Yoo, A.: Green, A.: Arunthamakun, J.: Wille, K. M.: Shaukat, T.: Singh, H.: Admon, A. J.: Semler, M. W.: Hernan, M. A.: Mueller, A. L.: Wang, W.: Leaf, D. E.: Walther, C. P.: Anumudu, S. J.: Kopecky, K. F.: Milligan, G. P.: McCullough, P. A.: Nguyen, T. D.: Shaefi, S.: Baskin, S. M.: Shankar, S.: Valencia, J. D.: Pannu, A.: Hayes, M. M.: Grandin, E. W.: Waikar, S. S.: Kibbelaar, Z. A.: Athavale, A. M.: Hart, P.: Upadhyay, S.: Vohra, I.: Oyintayo, A.: Rachoin, J. S.: Schorr, C. A.: Shea, L.: Edmonston, D. L.: Mosher, C. L.: Shehata, A. M.: Cohen, Z.: Allusson, V.: Bambrick-Santoyo, G.: Bhatti, N.: Mehta, B.: Williams, A.: Walters, P.: Go, R. C.: Rose, K. M.: Lisk, R.: Chan, L.: Coca, S. G.: Altman, D. R.: Saha, A.: Soh, H.: Wen, H. H.: Bose, S.: Leven, E. A.: Wang, J. G.: Mosoyan, G.: Nadkarni, G. N.: Pattharanitima, P.: Gallagher, E. J.: Friedman, A. N.: Guirguis, J.: Kapoor, R.: Meshberger, C.: Kelly, K. J.: Parikh, C. R.: Garibaldi, B. T.: Corona-Villalobos, C. P.: Wen, Y.: Menez, S.: Malik, R. F.: Cervantes, C. E.: Gautam, S. C.: Mallappallil, M. C.: Ouyang, J.: John, S.: Yap, E.: Melaku, Y.: Mohamed, I.: Bajracharya, S.: Puri, I.: Thaxton, M.: Bhattacharya, J.: Wagner, J.: Boudourakis, L.: Nguyen, H. B.: Ahoubim, A.: Thomas, L. F.: Sirganagari, D. R.: Guru, P. K.: Kashani, K.: Tehranian, S.: Zhou, Y.: Bergl, P. A.: Rodriguez, J.: Shah, J. A.: Gupta, M. S.: Kumar, P. N.: Lazarous, D. G.: Kassaye, S. G.: Melamed, M. L.: Mocerino, T. S. J. R.: Prudhvi, K.: Zhu, D.: Levy, R. V.: Azzi, Y.: Fisher, M.: Yunes, M.: Sedaliu, K.: Golestaneh, L.: Brogan, M.: Kumar, N.: Chang, M.: Thakkar, J.: Raichoudhury, R.: Athreya, A.: Farag, M.: Schenck, E. J.: Cho, S. J.: Plataki, M.: Alvarez-Mulett, S. L.: Gomez-Escobar, L. G.: Pan, D.: Lee, S.: Krishnan, J.: Whalen, W.: Charytan, D.: Macina, A.: Wu, B.: Modersitzki, F.: Leidner, A. S.: Martinez, C.: Kruser, J. M.: Wunderink, R. G.: Hodakowski, A. J.: Velez, J. C. Q.: Price-Haywood, E. G.: Matute-Trochez, L. A.: Hasty, A. E.: Mohamed, M. M. B.: Avasare, R. S.: Zonies, D.: Sise, M. E.: Newman, E. T.: Omar, S. A.: Pokharel, K. K.: Correa, S.: Kamal, O.: Yang, H.: Boateng, J. O.: Lee, M.: Strohbehn, I. A.: Li, J.: Redfern, R. E.: Cairl, N. S.: Naimy, G.: Abu-Saif, A.: Hall, D.: Bickley, L.: Rowan, C.: Madhani-Lovely, F.: Reiser, J.: Byun, J. J.: Vissing, A.: Kapania, E. M.: Post, Z.: Patel, N. P.: Hermes, J. M.: Sutherland, A. K.: Patrawalla, A.: Finkel, D. G.: Danek, B. A.: Arikapudi, S.: Paer, J. M.: Cangialosi, P.: Liotta, M.: Radbel, J.: Puri, S.: Sunderram, J.: Scharf, M. T.: Ahmed, A.: Berim, I.: Vatson, J. S.: Anand, S.: Levitt, J. E.: Garcia, P.: Boyle, S. M.: Song, R.: Arif, A.: Zhang, J.: Woo, S. H.: Deng, X.: Katz-Greenberg, G.: Senter, K.: Sharshir, M. A.: Rusnak, V. V.: Ali, M. I.: Peters, T.: Hughes, K.: Podoll, A. S.: Chonchol, M.: Sharma, S.: Burnham, E. L.: Rashidi, A.: Hejal, R.: Judd, E.: Latta, L.: Tolwani, A.: Albertson, T. E.: Adams, J. Y.: Chang, S. Y.: Beutler, R. M.: Schulze, C. E.: Macedo, E.: Rhee, H.: Liu, K. D.: Jotwani, V. K.: Koyner, J. L.: Kunczt, A.: Shah, C. V.: Jaikaransingh, V.: Toth-Manikowski, S. M.: Joo, M. J.: Lash, J. P.: Neyra, J. A.: Chaaban, N.: Elias, M.: Ahmad, Y.: Dy, R.: Iardino, A.: Au, E. H.: Sharma, J. H.: Sosa, M. A.: Taldone, S.: Contreras, G.: De La Zerda, D.: Fornoni, A.: Gershengorn, H. B.: Blakely, P.: Berlin, H.: Azam, T. U.: Shadid, H.: Pan, M.: Hayer, P. O.: Meloche, C.: Feroze, R.: Padalia, K. J.: Bitar, A.: Leya, J.:

Donnelly, J. P.: Flythe, J. E.: Tugman, M. J.: Chang, E. H.: Brown, B. R.: Leonberg-Yoo, A. K.: Spiardi, R. C.: Miano, T. A.: Roche, M. S.: Vasquez, C. R.: Bansal, A. D.: Ernecoff, N. C.: Kapoor, S.: Verma, S.: Chen, H.: Kovesdy, C. P.: Molnar, M. Z.: Azhar, A.: Hedayati, S. S.: Nadamuni, M. V.: Shastri, S.: Willett, D. L.: Short, S. A. P.: Renaghan, A. D.: Enfield, K. B.: Bhatraju, P. K.: Malik, A. B.: Vijayan, A.: Joy, C. M.: Li, T.: Goldberg, S.: Kao, P. F.: Goyal, N.: Faugno, A. J.: Schumaker, G. L.: Hsu, C. M.: Tariq, A.: Meyer, L.: Kshirsagar, R. K.: Jose, A.: Weiner, D. E.: Christov, M.: Griffiths, J.: Gupta, S.: Kapoor, A.: Chugh, S.: Wilson, P.: Arora, T.: Ugwuowo, U. Extracorporeal membrane oxygenation in patients with severe respiratory failure from COVID-19. *Intensive Care Medicine*. February 2021;doi:https://dx.doi.org/10.1007/s00134-020-06331-9

26. Butler AMB, Alexander: Sahrmann, John M.: Brookhart, M. Alan. Estimating the Effectiveness of Rotavirus Vaccine Schedules. *Epidemiology (Cambridge, Mass)*. 2021 Jul 01: 2022-01-27 2021;doi:http://dx.doi.org/10.1097/EDE.0000000000001363

27. Butler AML, J. B.: Reams, B. D.: Panozzo, C. A.: Brookhart, M. A. Study design and analytic approach for research on the effectiveness of childhood vaccine schedules. *Pharmacoepidemiology and Drug Safety*. August 2016;doi:http://dx.doi.org/10.1002/pds.4070

28. C. D. Mitnick RAW, C. Lu, C. A. Rodriguez, J. Bayona, M. C. Becerra, et al. Multidrug-resistant tuberculosis treatment failure detection depends on monitoring interval and microbiological method. *European Respiratory Journal*. 2016;doi:10.1183/13993003.00462-2016

29. Canfield S, Kemeter MJ, Febbo PG, Hornberger J. Balancing Confounding and Generalizability Using Observational, Real-world Data: 17-gene Genomic Prostate Score Assay Effect on Active Surveillance. *Rev Urol*. 2018;20(2):69-76. doi:10.3909/riu0799

30. Caniglia ECM, Eleanor J.: Hernán, Miguel A.: Shahn, Zach. Estimating optimal dynamic treatment strategies under resource constraints using dynamic marginal structural models. *Statistics in medicine*. 2021;doi:10.1002/sim.9107

31. Caporale JEP-R, A.: Beratarrechea, A. G.: Schulz-Hausmann, C. V.: Augustovski, F. A Comparison of 1-Year Treatment Costs in Patients with Type 2 Diabetes Following Initiation of Insulin Glargine or Insulin Detemir in Argentina. *Value Health Reg Issues*. Dec 2014;doi:10.1016/j.vhri.2014.02.007

32. Chaisson LHR, M.: Cantu, D.: Haller, B.: Millman, A. J.: Cattamanchi, A.: Davis, J. L. Impact of GeneXpert MTB/RIF assay on triage of respiratory isolation rooms for inpatients with presumed tuberculosis: a hypothetical trial. *Clin Infect Dis*. Nov-15 2014;doi:10.1093/cid/ciu620

33. Chakravarti PW, A.: Krikov, S.: Shao, N.: van der Laan, M. PIN68 Estimating Effects in Observational Real-World Data, From Target Trials to Targeted Learning: Example of Treating COVID-Hospitalized Patients. *Value in Health*. June 2021;doi:http://dx.doi.org/10.1016/j.jval.2021.04.1256

34. Challa APN, X.: Garrison, E. A.: Van Driest, S. L.: Bastarache, L. M.: Lippmann, E. S.: Lavieri, R. R.: Goldstein, J. A.: Aronoff, D. M. Clinical trial emulation can identify new opportunities to enhance the regulation of drug safety in pregnancy. *medRxiv*. 2021;doi:https://dx.doi.org/10.1101/2021.11.12.21266269

35. Chang P-YW, Wei Ting: Wu, Wei-Lun: Chang, Hui-Chin: Chen, Chen Huan: Tsai, Yi Wen: Chiou, Shih-Hwa: Lip, Gregory Y. H.: Cheng, Hao Min: Chiang, Chern-En. Oral Anticoagulation Timing in Patients with Acute Ischaemic Stroke and Atrial Fibrillation. *Thrombosis and haemostasis*. 2021;doi:10.1055/a-1669-4987

© 2023 Hansford HJ et al. *JAMA Network Open.*

36. Chen X. Causal Inference and Prediction on Observational Data with Survival Outcomes. *NA*. 2020;doi:NA

37. Chi-Shin WY, Albert C.: Chang, Shu-Sen: Chang, Chia-Ming: Liu, Yi-Hung: Liao, Shih-Cheng: Tsai, Hui-Ju. Validation of Machine Learning-Based Individualized Treatment for Depressive Disorder Using Target Trial Emulation. *Journal of personalized medicine*. 2021 Dec 07: 2022-01-28 2021;doi:http://dx.doi.org/10.3390/jpm11121316

38. Conner SCL, Sara: Lunetta, Kathryn L.: Casas, Juan P.: Lubitz, Steven A.: Ellinor, Patrick T.: Anderson, Christopher D.: Huang, Qiuxi: Coleman, Justin: White, Wendy B.: Benjamin, Emelia J.: Trinquart, Ludovic. Refining the Association Between Body Mass Index and Atrial Fibrillation: G-Formula and Restricted Mean Survival Times. *Journal of the American Heart Association*. 2019;doi:10.1161/jaha.119.013011

39. Cuzin LP, Pascal: Allavena, Clotilde: Katlama, Christine: Cotte, Laurent: Cheret, Antoine: Cabié, André: Rey, David: Chirouze, Catherine: Bani-Sadr, Firouze: Flandre, Philippe: Dat', Aids Study group. Comparative Effectiveness of First Antiretroviral Regimens in Clinical Practice Using a Causal Approach. *Medicine*. September 2015: 2022-01-27 2015;doi:http://dx.doi.org/10.1097/MD.0000000000001668

40. D'Amico G, Abraldes JG, Rebora P, Valsecchi MG, Garcia-Tsao G. Ordinal Outcomes Are Superior to Binary Outcomes for Designing and Evaluating Clinical Trials in Compensated Cirrhosis. *Hepatology*. 2020;72(3):1029-1042. doi:https://doi.org/10.1002/hep.31070

41. D'Amico G, Abraldes JG, Rebora P, Valsecchi MG, Garcia‐Tsao G. Ordinal outcomes are superior to binary outcomes for designing and evaluating clinical trials in compensated cirrhosis. *Hepatology*. 2020;72(3):1029-1042.

42. Danaei G, Pan, An, Hu, Frank B. and Hernán, Miguel A. Hypothetical Midlife Interventions in Women and Risk of Type 2 Diabetes. *Epidemiology*. 2013;doi:10.1097/EDE.0b013e318276c98a

43. Ferguson ND, Scales DC, Pinto R, et al. Integrating mortality and morbidity outcomes: using quality-adjusted life years in critical care trials. *Am J Respir Crit Care Med*. Feb 1 2013;187(3):256-61. doi:10.1164/rccm.201206-1057OC

44. Garcia-Aymerich J, Varraso R, Danaei G, Camargo CA, Jr., Hernán MA. Incidence of adult-onset asthma after hypothetical interventions on body mass index and physical activity: an application of the parametric g-formula. *Am J Epidemiol*. Jan 1 2014;179(1):20-6. doi:10.1093/aje/kwt229

45. Ghione P, Palomba ML, Patel AR, et al. Comparative effectiveness of ZUMA-5 (axi-cel) vs SCHOLAR-5 external control in relapsed/refractory follicular lymphoma. *Blood*. 2022;140(8):851-860. doi:10.1182/blood.2021014375

46. Giannakeas V, Sopik V, Narod S. A Validation of Methods for the Evaluation of Observational Studies of Screening Mammography: An Exploratory Analysis Based on Simulating Screening Cohorts. *Clin Epidemiol*. 2020;12:1161-1169. doi:10.2147/clep.S267584

47. Gottenberg J-EM, Jacques: Perrodeau, Elodie: Bardin, Thomas: Combe, Bernard: Dougados, Maxime: Flipo, René-Marc: Saraux, Alain: Schaeverbeke, Thierry: Sibilia, Jean: Soubrier, Martin: Vittecoq, Olivier: Baron, Gabriel: Constantin, Arnaud: Ravaud, Philippe: Mariette, Xavier. Comparative effectiveness of rituximab, abatacept, and tocilizumab in adults with rheumatoid arthritis and inadequate response to TNF inhibitors: prospective cohort study. *BMJ (Clinical research ed)*. 2019;doi:10.1136/bmj.l67

© 2023 Hansford HJ et al. *JAMA Network Open.*

48. Hao YH, W. C.: Parzynski, C. S.: Lobetti Bodoni, C.: Hampson, L.: Masood, A.: Rhodes, W.: Wu, W. H. Utilization of External Control Arm to Contextualize Clinical Efficacy of Tisagenlecleucel Treated Among Patients with Relapsed/Refractory Follicular Lymphoma from the Single-Arm Elara Trial. *Blood*. 23-Nov 2021;doi:https://dx.doi.org/10.1182/blood-2021-152685

49. Harlow AF, Stokes AC, Brooks DR, et al. Prospective association between e-cigarette use frequency patterns and cigarette smoking abstinence among adult cigarette smokers in the United States. *Addiction (Abingdon, England)*. 2022;NA(NA):NA-NA. doi:10.1111/add.16009

50. Hazzouri AZAJ, Neal: Grasset, Leslie: Kaiser, Paulina: Kezios, Katrina L.: Calonico, Sebastian: Glymour, M. Maria: Hirsch, Calvin H.: Arnold, Alice M.: Varadhan, Ravi: Opoodden, Michelle C. Cannot retrieve FT - Statins and cognitive decline in the Cardiovascular Health Study: A comparison of different analytical approaches. *The journals of gerontology Series A, Biological sciences and medical sciences*. 2021;doi:10.1093/gerona/glab220

51. Henry DA, Jones MA, Stehlik P, Glasziou PP. Effectiveness of COVID‐19 vaccines: findings from real world studies. *The Medical Journal of Australia*. 2021;215(4):149.

52. Hernán MA, Robins J. Observational Studies Analyzed Like Randomized Trials and Vice Versa.
Methods in Comparative Effectiveness Research. 1st ed. Chapman and Hall/CRC; 2017:22.

53. Högnäs RSB, Maarten J.: Högnäs, Ulf: Blomqvist, Sandra: Westerlund, Hugo: Hanson, Linda Magnusson. It's giving me the blues: A fixed-effects and g-formula approach to understanding job insecurity, sleep disturbances, and major depression. *Social science & medicine (1982)*. 2022;doi:10.1016/j.socscimed.2022.114805

54. Hu A-MS, Zhi-Ming: Zhang, Zhong-Jun: Li, Hui-Ping. Comparative Efficacy of Fentanyl and Morphine in Patients with or At Risk for Acute Respiratory Distress Syndrome: A Propensity Score-Matched Cohort Study. *Drugs in R&D*. 2021;doi:http://dx.doi.org/10.1007/s40268-021-00338-3

55. Huang BM, E. M.: Chen, C.: Guo, J.: Lovell, D. J.: Brunner, H. I. THU0547 Real-world effectiveness of early aggressive treatment with biological disease-modifying antirheumatic drugs for the treatment of newly diagnosed polyarticular form of juvenile idiopathic arthritis. *Annals of the Rheumatic Diseases*. 2018;doi:http://dx.doi.org/10.1136/annrheumdis-2018-eular.3705

56. Jafarzadeh SRT, Benjamin S.: Marschall, Jonas: Fraser, Victoria J.: Gill, Jeff: Warren, David K. Quantifying the improvement in sepsis diagnosis, documentation, and coding: the marginal causal effect of year of hospitalization on sepsis diagnosis. *Annals of epidemiology*. 2015;doi:10.1016/j.annepidem.2015.10.008

57. Jain PS, Claudia K.: Rexrode, Kathryn M.: Manson, JoAnn E.: Robins, James M.: Hernán, Miguel A.: Danaei, Goodarz. Hypothetical Lifestyle Strategies in Middle-Aged Women and the Long-Term Risk of Stroke. *Stroke*. 2020;doi:10.1161/strokeaha.119.026761

58. Jelinek TM, Vladimir: Pour, Lud_k: Spicka, Ivan: Mina_ík, Ji_í: Gregora, Ev_en: Kessler, Petr: S_kora, Michal: Fra_ková, Hana: Adamova, Dagmar: Wrobel, Marek: Mikula, Peter: Jarkovsk_, Ji_í: Diels, Joris: Gatopoulou, Xenia: Veselá, _árka: Besson, Hervé: Bro_ová, Lucie: Ito, Tetsuro: Hájek, Roman. Adjusted comparison of daratumumab monotherapy versus real-world historical control data from the Czech Republic in heavily pretreated and highly refractory

© 2023 Hansford HJ et al. *JAMA Network Open.*

multiple myeloma patients. *Current medical research and opinion*. 2018;doi:10.1080/03007995.2017.1410121

59. Jemielita TW, Linnea: Fox, Claire: Salomonsson, Stina: Liaw, Kai-Li: Pettersson, Andreas. Replication of Oncology Randomized Trial Results using Swedish Registry Real World-Data: A Feasibility Study. *Clinical pharmacology and therapeutics*. 2021;doi:http://dx.doi.org/10.1002/cpt.2424

60. Karthikesalingam AW, Anders: Holt, Peter J. E.: Vidal-Diez, Alberto: Brownrigg, Jack: Shpitser, Ilya: Björck, Martin: Thompson, Matt M.: Mani, Kevin. Comparison of long-term mortality after ruptured abdominal aortic aneurysm in England and Sweden. *The British journal of surgery*. 2015;doi:10.1002/bjs.10049

61. Kim YT, Yuxi: Yang, Jianxiao: Huser, Vojtech: Jin, Peng: Lambert, Christophe G.: Park, Hojun: You, Seng Chan: Park, Rae Woong: Rijnbeek, Peter R.: Van Zandt, Mui: Reich, Christian: Vashisht, Rohit: Wu, Yonghui: Duke, Jon: Hripcsak, George: Madigan, David: Shah, Nigam H.: Ryan, Patrick B.: Schuemie, Martijn J.: Suchard, Marc A. Comparative safety and effectiveness of alendronate versus raloxifene in women with osteoporosis. *Scientific reports*. 2020;doi:http://dx.doi.org/10.1038/s41598-020-68037-8

62. Kishimoto T, Churiki M, Miyazato T, Yamashiro A, Nagasawa Y, Shokita H. Association between lifestyle and metabolic syndrome incidence of workers in northern Okinawa, Japan: A cohort study. *Preventive Medicine Reports*. December 2022;30 (no pagination)101995. doi:https://dx.doi.org/10.1016/j.pmedr.2022.101995

63. Kuehne FJ, B.: Conrads-Frank, A.: Bundo, M.: Arvandi, M.: Endel, F.: Popper, N.: Endel, G.: Urach, C.: Gyimesi, M.: Murray, E. J.: Danaei, G.: Gaziano, T. A.: Pandya, A.: Siebert, U. Guidance for a causal comparative effectiveness analysis emulating a target trial based on big real world evidence: When to start statin treatment. *Journal of Comparative Effectiveness Research*. 2019;doi:http://dx.doi.org/10.2217/cer-2018-0103

64. Li Y, Wang Q, Liang S, et al. Effect of Switching Antiretroviral Treatment Regimen in Patients With Drug-Resistant HIV-1 Infection: Retrospective Observational Cohort Study. *JMIR public health and surveillance*. 2022;8(6):e33429-e33429. doi:10.2196/33429

65. Lyu B, Hwang YJ, Selvin E, et al. Glucose-Lowering Agents and the Risk of Hypoglycemia: a Real-world Study. *Journal of general internal medicine*. 2022;NA(NA):NA-NA. doi:10.1007/s11606-022-07726-8

66. Mack CDP, Alessandra: Kelly, Kim: Irwin, Debra E.: Maislin, Greg: Jones, John: Wester, Tawana: Zura, Robert. Breaking Barriers: Studying Fracture Healing in the BONES Program. *Journal of orthopaedic trauma*. 2021 Mar 01: 2022-01-27 2021;doi:10.1002/14651858.CD008579. AETNA. Bone Growth Stimulators. 2021. [www.aetna.com]. Available at: http://www.aetna.com/cpb/medical/data/300_399/0343.html. Accessed January 6, 2021. BlueCross BlueShield BlueCare Network of Michigan. Bone Growth Stimulation: Ultrasound Accelerated Fracture Healing Device. 2020. [www.bcbsm.com]. Available at: https://www.bcbsm.com/amslibs/content/dam/public/mpr/mprsearch/pdf/2030582.pdf. Accessed January 6, 2021. US Food and Drug Administration (FDA). Framework for the FDA's real world evidence program. 2018. [www.fda.gov]. Available at: https://www.fda.gov/media/120060/download. Accessed May 16, 2020. Boudreau DM, Yu O, Spangler L, et al. Accuracy of ICD-9 codes to identify nonunion and malunion and developing

© 2023 Hansford HJ et al. *JAMA Network Open.*

algorithms to improve case-finding of nonunion and malunion. Bone. 2013;52:596–601. Hernandez RK, Do TP, Critchlow CW, et al. Patient-related risk factors for fracture-healing complications in the United Kingdom general Practice research database. Acta Orthop. 2012;83:653–660. Zura R, Xiong Z, Einhorn T, et al. Epidemiology of fracture in 18 human bones. JAMA Surg. 2016;151:e162775. Irwin DE, Kelly K, Winer I, et al. Methodologies for validation of diagnoses in real-world data: BONES–a case study. J Ortho Trauma. 2021;35 (suppl 1): S28–S32. Chronic conditions data warehouse (CCDW). 2020. [www.ccwdata.org]. Available at: https://www2.ccwdata.org/web/guest/home/. Accessed October 16, 2020. Rubin D. For objective causal inference, design trumps analysis. Annappl Stat. 2008;2:808–840. Campbell G, Yue LQ. Statistical innovations in the medical device world sparked by the FDA. J Biopharm Stat. 2016;26:3–16. Yue LQ. Regulatory considerations in the design of comparative observational studies using propensity scores. J Biopharm Stat. 2012;22:1272–1279. Mahendraratnam N, Mercon K, Eckert J, et al. Adding real-world evidence to a totality of evidence approach for evaluating marketed product effectiveness. 2019. [www.healthpolicy.duke.edu]. Available at: https://healthpolicy.duke.edu/publications/adding-real-world-evidence-totality-evidence-approach-evaluating-marketed-product. Accessed October 19, 2020. Mahendraratnam N, Eckert J, Mercon K, et al. Understanding the need for non-interventional studies using secondary data to generate real-world evidence for regulatory decision making and demonstrating their credibility. 2019. [https://healthpolicy.duke.edu]. Available at: https://healthpolicy.duke.edu/publications/understanding-need-non-interventional-studies-using-secondary-data-generate-real-world. Accessed October 19, 2020. Krumholz H. Opinion: it's your right to see your medical records. It shouldn't be this hard to do. 2020. [www.npr.org]. Available at: https: /www.npr.org/sections/health-shots/2019/08/28/754725843/opinion-its-your-right-to-see-your-medical-records-it-shouldn-t-be-this-hard-to-. Accessed October 19, 2020. Zura R, Irwin DE, Mack CD. Real-world evidence: a primer. J Ortho Trauma. 2021; 35 (suppl 1): S1–S5.: http://dx.doi.org/10.1097/BOT.0000000000002035

67. Massaad EA. Validation of Clinical Prediction Models and Study of the Role of Modern Therapies Using Real-World Data in Spine Oncology. *Journal*. 2021;

68. McGinty EET, K. N.: Barry, C. L.: Bicket, M. C.: Rutkow, L.: Stuart, E. A. Protocol: mixed-methods study of how implementation of US state medical cannabis laws affects treatment of chronic non-cancer pain and adverse opioid outcomes. *Implementation science : IS*. 7-Jan 2021;doi:https://dx.doi.org/10.1186/s13012-020-01071-2

69. Medenwald D, Vordermark D, Dietzel CT. Early Mortality of Prostatectomy vs. Radiotherapy as a Primary Treatment for Prostate Cancer: A Population-Based Study From the United States and East Germany. Original Research. *Frontiers in Oncology*. 2020-January-17 2020;9doi:10.3389/fonc.2019.01451

70. Mokhayeri Y, Nazemipour M, Mansournia MA, Naimi AI, Kaufman JS. Does weight mediate the effect of smoking on coronary heart disease? Parametric mediational g-formula analysis. *PloS one*. 2022;17(1):e0262403-e0262403. doi:10.1371/journal.pone.0262403

71. Moon SL, Hae Young: Jang, Jieun: Park, Sue K. Association Between Angiotensin II Receptor Blockers and the Risk of Lung Cancer Among Patients With Hypertension From the Korean National Health Insurance Service-National Health Screening Cohort. *Journal of preventive medicine and public health = Yebang Uihakhoe chi*. 2020;doi:10.3961/jpmph.20.405

© 2023 Hansford HJ et al. *JAMA Network Open.*

72. Moreno-Betancur MM, P.: Becker, D.: Patton, G. C.: Carlin, J. B. Mediation effects that emulate a target randomised trial: Simulation-based evaluation of ill-defined interventions on multiple mediators. *Statistical Methods in Medical Research*. June 2021;doi:http://dx.doi.org/10.1177/0962280221998409
73. Moriarty F, Thompson W, Boland F. Methods for evaluating the benefit and harms of deprescribing in observational research using routinely collected data. *Res Social Adm Pharm*. Feb 2022;18(2):2269-2275. doi:10.1016/j.sapharm.2021.05.007
74. Moriarty FT, W.: Boland, F. Methods for evaluating the benefit and harms of deprescribing in observational research using routinely collected data. *Research in social & administrative pharmacy : RSAP*. 1-Feb 2022;doi:https://dx.doi.org/10.1016/j.sapharm.2021.05.007
75. Moura LM, Westover MB, Kwasnik D, Cole AJ, Hsu J. Causal inference as an emerging statistical approach in neurology: an example for epilepsy in the elderly. *Clinical epidemiology*. 2016;9:9-18. doi:10.2147/CLEP.S121023
76. Murray EJ, Marshall BDL, Buchanan AL. Emulating Target Trials to Improve Causal Inference From Agent-Based Models. *American Journal of Epidemiology*. 2021;190(8):1652-1658. doi:10.1093/aje/kwab040
77. Neugebauer R, Fireman B, Roy JA, Raebel MA, Nichols GA, O'Connor PJ. Super learning to hedge against incorrect inference from arbitrary parametric assumptions in marginal structural modeling. *J Clin Epidemiol*. Aug 2013;66(8 Suppl):S99-109. doi:10.1016/j.jclinepi.2013.01.016
78. O'Sullivan DE, Cheung WY, Boyne DJ, et al. Treatment patterns and survival outcomes of early-onset colorectal cancer patients in Alberta, Canada: a population-based study. *Cancer treatment and research communications*. 2022;32(NA):100585-100585. doi:10.1016/j.ctarc.2022.100585
79. Ön BIV, Xavier: Berger, Ursula: Sabaté, Mònica: Ballarín, Elena: Maisterra, Olga: San-Jose, Antonio: Ibáñez, Luisa. Antidepressant use and stroke or mortality risk in the elderly. *European journal of neurology*. 2021;doi:10.1111/ene.15137
80. Piccini JP. The importance of the design of observational studies in comparative effectiveness research: Lessons from the GARFIELD-AF and ORBIT-AF registries. *The American Heart Journal*. 2022;doi:http://dx.doi.org/10.1016/j.ahj.2021.09.003
81. Prats-Uribe AT, M.: Villacampa, J. M.: Aguero, A.: Garcia-Bastida, C.: Tato, J. I.: Rodriganez, L.: Holguera, V. D.: Hernandez-Garcia, E.: Poletti, D.: Simonetti, G.: Villarraga, V.: Meler-Claramonte, C.: Sanchez Barrueco, A.: Chiesa-Estomba, C.: Casasayas, M.: Parente-Arias, P.: Mata-Castro, N.: Rello, J.: Castro, P.: Prieto-Alhambra, D.: Vilaseca, I.: Aviles-Jurado, F. X. Timing of elective tracheotomy and duration of mechanical ventilation among patients admitted to intensive care with severe COVID-19: A multicenter prospective cohort study. *Head & neck*. 1-Dec 2021;doi:https://dx.doi.org/10.1002/hed.26863
82. Prosperi M, Ghosh S, Chen Z, et al. Causal AI with Real World Data: Do Statins Protect from Alzheimer's Disease Onset? presented at: 2021 5th International Conference on Medical and Health Informatics; 2021; Kyoto, Japan. https://doi.org/10.1145/3472813.3473206
83. Rassen JA, Murk W, Schneeweiss S. Real-world evidence of bariatric surgery and cardiovascular benefits using electronic health records data: A lesson in bias. *Diabetes, Obesity and Metabolism*. 2021;23(7):1453-1462. doi:https://doi.org/10.1111/dom.14338

© 2023 Hansford HJ et al. *JAMA Network Open.*

84. Reeves BC, Scott LJ, Taylor J, et al. Effectiveness of Community versus Hospital Eye Service follow-up for patients with neovascular age-related macular degeneration with quiescent disease (ECHoES): a virtual non-inferiority trial. *BMJ Open*. 2016;6(7):e010685-e010685. doi:10.1136/bmjopen-2015-010685
85. Ren JL, Guangyou: Zhang, Liju: Zhang, Na: Ren, Juan. Hypothetical Interventions for Falls Among Older Adults: An Application of the Parametric G-Formula. *Frontiers in medicine*. 2021;doi:10.3389/fmed.2021.732136
86. Requena GW, A.: Williams, R.: Dedman, D.: Quint, J. K.: Murray-Thomas, T.: Pimenta, J. M. Feasibility of using Clinical Practice Research Datalink data to identify patients with chronic obstructive pulmonary disease to enrol into real-world trials. *Pharmacoepidemiol Drug Saf*. Apr 2021;doi:10.1002/pds.5188
87. Richardson KM, Katharina: Loke, Yoon K.: Steel, Nicholas: Fox, Chris: Grossi, Carlota M.: Bennett, Kathleen: Maidment, Ian: Boustani, Malaz: Matthews, Fiona E.: Myint, Phyo K.: Campbell, Noll L.: Brayne, Carol: Robinson, Louise: Savva, George M. History of Benzodiazepine Prescriptions and Risk of Dementia: Possible Bias Due to Prevalent Users and Covariate Measurement Timing in a Nested Case-Control Study. *American journal of epidemiology*. 2019;doi:10.1093/aje/kwz073
88. Roberts MHM, Douglas W.: Petersen, Hans. Comparative Causal Analysis of the Effects of Long-Acting Muscarinic Antagonist Versus No Long-Acting Bronchodilator Use on Readmission or Mortality After Hospitalization for Chronic Obstructive Pulmonary Disease. *Drugs - real world outcomes*. March 2020: 2022-01-27 2020;doi:http://dx.doi.org/10.1007/s40801-019-00171-w
89. Rostanski SKS, Zachary: Elkind, Mitchell S. V.: Liberman, Ava L.: Marshall, Randolph S.: Stillman, Joshua I.: Williams, Olajide: Willey, Joshua Z. Door-to-Needle Delays in Minor Stroke: A Causal Inference Approach. *Stroke*. 2017;doi:10.1161/strokeaha.117.017386
90. Sarayani A, Brown JD, Goodin AJ, et al. A Pharmacoepidemiologic Approach to Evaluate Real-world Effectiveness of Hormonal Contraceptives in the Presence of Drug-drug Interactions. *Epidemiology (Cambridge, Mass)*. 2021;32(2):268-276. doi:10.1097/EDE.0000000000001302
91. Schnitzer ME, Guerra SF, Longo C, Blais L, Platt RW. A potential outcomes approach to defining and estimating gestational age-specific exposure effects during pregnancy. *Stat Methods Med Res*. Feb 2022;31(2):300-314. doi:10.1177/09622802211065158
92. Shin H, Schneeweiss S, Glynn RJ, Patorno E. Evolving channeling in prescribing SGLT-2 inhibitors as first-line treatment for type 2 diabetes. *Pharmacoepidemiology and drug safety*. 2022;31(5):566-576. doi:10.1002/pds.5406
93. Suchard MAS, Martijn J.: Krumholz, Harlan M.: You, Seng Chan: Chen, Ruijun: Pratt, Nicole L.: Reich, Christian G.: Duke, Jon: Madigan, David: Hripcsak, George: Ryan, Patrick B. Comprehensive comparative effectiveness and safety of first-line antihypertensive drug classes: a systematic, multinational, large-scale analysis. *Lancet (London, England)*. 2019;doi:10.1016/s0140-6736(19)32317-7
94. Suissa S. Single-arm Trials with Historical Controls: Study Designs to Avoid Time-related Biases. *Epidemiology (Cambridge, Mass)*. January 2021: 2022-01-27 2021;doi:http://dx.doi.org/10.1097/EDE.0000000000001267

© 2023 Hansford HJ et al. *JAMA Network Open.*

95. Szmulewicz AGB, Brian T.: Levin, Raisa: Huybrechts, Krista F. Risk of Overdose Associated With Co-prescription of Antipsychotics and Opioids: A Population-Based Cohort Study. *Schizophrenia bulletin*. 2021;doi:10.1093/schbul/sbab116
96. Tan KB, Jonathan: Segal, Brian D.: Bellomo, Lawrence: Nussbaum, Nate: Tucker, Melisa: Torres, Aracelis Z.: Bennette, Carrie: Capra, William B.: Curtis, Melissa D.: Miksad, Rebecca A. Emulating Control Arms for Cancer Clinical Trials Using External Cohorts Created From Electronic Health Record-Derived Real-World Data. *Clinical pharmacology and therapeutics*. 2021;doi:10.1002/cpt.2351
97. Tardif I, Guénette L, Zongo A, Demers É, Lunghi C. Depression and the risk of hospitalization in type 2 diabetes patients: A nested case-control study accounting for non-persistence to antidiabetic treatment. *Diabetes & metabolism*. 2022;48(4):101334-101334. doi:10.1016/j.diabet.2022.101334
98. Taylor PT, D.: Lee, A. Y.: Mueller, P. L.: Schwartz, R.: Olvera-Barrios, A.: Warwick, A.: Patel, P. J.: Herren, T.: Egan, C.: Tufail, A. Emulated trial comparing the effectiveness of Bevacuzimab vs Aflibercept using an external control arm. *Investigative Ophthalmology and Visual Science Conference: Annual Meeting Association for Research in Vision and Ophthalmology, ARVO*. 2021;
99. Toh SM, JoAnn E. An Analytic Framework for Aligning Observational and Randomized Trial Data: Application to Postmenopausal Hormone Therapy and Coronary Heart Disease. *Statistics in biosciences*. 2012;doi:10.1007/s12561-012-9073-6
100. Trujillo-Santos J, Farge-Bancel D, Pedrajas JM, et al. Enoxaparin versus dalteparin or tinzaparin in patients with cancer and venous thromboembolism: The RIETECAT study. *Research and practice in thrombosis and haemostasis*. 2022;6(4):e12736-NA. doi:10.1002/rth2.12736
101. Tsuzuki SH, Kayoko: N, Matsunaga: Terada, Mari: Suzuki, Satoshi: Ohtsu, Hiroshi: Asai, Yusuke: K, Kitajima: Saito, Sho: Uemura, Yukari: T, Shibata: Kondo, Masashi: K, Izumi: Hojo, Masayuki: Mizoue, Tetsuya: K, Yokota: F, Nakamura-Uchiyama: F, Saito: Sugiura, Wataru: Ohmagari, Norio. Efficacy of remdesivir in Japanese patients hospitalised with COVID-19: A large observational study using the COVID-19 Registry Japan. *NA*. 2021;doi:10.1101/2021.03.09.21253183
102. Weberpals JB, T.: Davies, J.: Schmich, F.: Ruttinger, D.: Theis, F. J.: Bauer-Mehren, A. Deep learning-based propensity scores for confounding control in comparative effectiveness research: A large-scale, real-world data study. *Epidemiology*. 2021;doi:http://dx.doi.org/10.1097/EDE.0000000000001338
103. Wharton JM, Piccini JP, Koren A, Huse S, Ronk CJ. Comparative Safety and Effectiveness of Sotalol Versus Dronedarone After Catheter Ablation for Atrial Fibrillation. *Journal of the American Heart Association*. 2022;11(3):e020506-NA. doi:10.1161/jaha.120.020506
104. Wu C-S, Yang AC, Chang S-S, et al. Validation of machine learning-based individualized treatment for depressive disorder using target trial emulation. *Journal of personalized medicine*. 2021;11(12):1316.
105. Zash RC, E.: Mayondi, G.: Diseko, M.: Mabuta, J.: Jacobson, D.: Johnson, K.: Mmalane, M.: Makhema, J.: Lockman, S.: Shapiro, R. The risk of gestational hypertension with use of dolutegravir at conception. *Topics in Antiviral Medicine*. March 2021;

106. Zhang YL, Li-An: Starkopf, Liis: Chen, Jie: Wang, William. Estimation of causal effect in integrating randomized clinical trial and observational data - An example application to cardiovascular outcome trial. *Contemporary clinical trials*. 2021;doi:10.1016/j.cct.2021.106492

Post-hoc exclusions - 'Implicit' target trial emulation

107. Hripcsak G, Suchard MA, Shea S, et al. Comparison of Cardiovascular and Safety Outcomes of Chlorthalidone vs Hydrochlorothiazide to Treat Hypertension. *JAMA Internal Medicine*. 2020;180(4):542-551. doi:10.1001/jamainternmed.2019.7454

108. Na EDC, Sung Woon: Ahn, Eun Hee: Jung, Sang Hee: Kim, Young Ran: Jung, Inkyung: Cho, Hee Young. Pregnancy outcomes of elective induction in low-risk term pregnancies: A propensity-score analysis. *Medicine*. 2019;doi:10.1097/md.0000000000014284

109. Naglich AC, Bozeman S, Brown ES, Adinoff B. Effect of Selective Serotonin Reuptake Inhibitors on Healthcare Utilization in Patients with Post-Traumatic Stress Disorder and Alcohol Use Disorder. *Alcohol Alcohol*. Jul 1 2019;54(4):428-434. doi:10.1093/alcalc/agz045

110. Patorno E, Schneeweiss S, Gopalakrishnan C, Martin D, Franklin JM. Using Real-World Data to Predict Findings of an Ongoing Phase IV Cardiovascular Outcome Trial: Cardiovascular Safety of Linagliptin Versus Glimepiride. *Diabetes Care*. Dec 2019;42(12):2204-2210. doi:10.2337/dc19-0069

111. You SC, Krumholz HM, Suchard MA, et al. Comprehensive Comparative Effectiveness and Safety of First-Line β-Blocker Monotherapy in Hypertensive Patients. *Hypertension*. 2021;77(5):1528-1538. doi:doi:10.1161/HYPERTENSIONAHA.120.16402

Abstract / Protocol / Other secondary publication (92)

112. Abu-Raddad LJC, Hiam: Ayoub, Houssein H.: AlMukdad, Sawsan: Tang, Patrick: Hasan, Mohammad R.: Coyle, Peter: Yassine, Hadi M.: Al-Khatib, Hebah A.: Smatti, Maria K.: Al-Kanaani, Zaina: Al-Kuwari, Einas: Jeremijenko, Andrew: Kaleeckal, Anvar Hassan: Latif, Ali Nizar: Shaik, Riyazuddin Mohammad: Abdul-Rahim, Hanan F.: Nasrallah, Gheyath K.: Al-Kuwari, Mohamed Ghaith: Butt, Adeel A.: Al-Romaihi, Hamad Eid: Al-Thani, Mohamed H.: Al-Khal, Abdullatif: Bertollini, Roberto. Effectiveness of BNT162b2 and mRNA-1273 COVID-19 boosters against SARS-CoV-2 Omicron (B.1.1.529) infection in Qatar. *NA*. 2022;doi:10.1101/2022.01.18.22269452

113. Amiri-Kordestani L, Gao X, Baxi S, et al. Generating real-world externa comparators for randomized clinical trials (RCTs) in metastatic breast cancer (mBC) using electronic health records (EHRs). Conference Abstract. *Cancer Research Conference*. 2021;82(4 SUPPL)doi:https://dx.doi.org/10.1158/1538-7445.SABCS21-P2-11-05

114. Andrew NC, L.: Kim, J.: Cadilhac, D.: Sundararajan, V.: Thrift, A.: Lannin, N.: Nelson, M.: Srikanth, V.: Kilkenny, M. Incentivising primary care practitioners to provide comprehensive chronic disease management improves survival following stroke: A target trial approach using linked registry data. *International Journal of Stroke*. October 2021;doi:http://dx.doi.org/10.1177/17474930211041949

115. Birdthistle I. Must the evaluation of complex interventions be complex? learning from the impact evaluation of dreams. *Sexually Transmitted Infections*. July 2019;doi:http://dx.doi.org/10.1136/sextrans-2019-sti.17

© 2023 Hansford HJ et al. *JAMA Network Open.*

116. Boyne DJC, W. Y.: Hilsden, R. J.: Sajobi, T. T.: Batra, A.: Friedenreich, C. M.: Brenner, D. R. Association of a Shortened Duration of Adjuvant Chemotherapy with Overall Survival among Individuals with Stage III Colon Cancer. *JAMA Network Open*. 30-Mar 2021;doi:http://dx.doi.org/10.1001/jamanetworkopen.2021.3587

117. Cain LES, Michael S.: Petersen, Maya L.: May, Margaret T.: Ingle, Suzanne M.: Logan, Roger: Robins, James M.: Abgrall, Sophie: Shepherd, Bryan E.: Deeks, Steven G.: Gill, M. John: Touloumi, Giota: Vourli, Georgia: Dabis, François: Vandenhende, Marie-Anne: Reiss, Peter: van Sighem, Ard: Samji, Hasina: Hogg, Robert S.: Rybniker, Jan: Sabin, Caroline A.: Jose, Sophie: del Amo, Julia: Moreno, Santiago: Rodriguez, Benigno: Cozzi-Lepri, Alessandro: Boswell, Stephen L.: Stephan, Christoph: Pérez-Hoyos, Santiago: Jarrín, Inma: Guest, Jodie L.: Monforte, Antonella d'Arminio: Antinori, Andrea: Moore, Richard D.: Campbell, Colin N. J.: Casabona, Jordi: Meyer, Laurence: Seng, Rémonie: Phillips, Andrew N.: Bucher, Heiner C.: Egger, Matthias: Mugavero, Michael J.: Haubrich, Richard: Geng, Elvin: Olson, Ashley: Eron, Joseph J.: Napravnik, Sonia: Kitahata, Mari M.: Van Rompaey, Stephen E.: Teira, Ramón: Justice, Amy C.: Tate, Janet P.: Costagliola, Dominique: Sterne, Jonathan A. C.: Hernán, Miguel A. Using observational data to emulate a randomized trial of dynamic treatment-switching strategies: an application to antiretroviral therapy. *International journal of epidemiology*. 2015;doi:10.1093/ije/dyv295

118. Charpignon M-LV-L, Bella: Zhang, B.: Magdomo, C.: Su, Bowen: Evans, Kyle: Rodriguez, Steven: Sololov, A.: Boswell, Sarah A.: Sheu, Yi-Han: Somai, M.: Middleton, Lefkos T.: Hyman, B. T.: Betensky, Rebecca A.: Finkelstein, S. N.: Welsch, R. E.: Tzoulaki, Ioanna: Blacker, Deborah: Das, Saumya: Albers, Mark W. Drug repurposing of metformin for Alzheimer's disease: Combining causal inference in medical records data and systems pharmacology for biomarker identification. *NA*. 2021;doi:10.1101/2021.08.10.21261747

119. Chase DP, J. A.: Gupta, D.: Kalilani, L.: Woodward, T.: Gonzalez-Martin, A. Disease progression in patients with ovarian cancer who received first-line maintenance therapy or active surveillance, a us realworld analysis. *International Journal of Gynecological Cancer*. October 2021;doi:https://dx.doi.org/10.1136/ijgc-2021-ESGO.455

120. Chen J, Diouf I, Taylor BV, Kalincik T, van der Mei I. Superior effects of natalizumab versus other DMTs on patient-reported outcomes in people with multiple sclerosis. *Journal of neurology, neurosurgery, and psychiatry*. 2022;93(10):1120-1127. doi:10.1136/jnnp-2022-329223

121. Dahabreh IJN, J.: Zullo, A. R.: Crown, W. H.: Shah, N. D.: Inker, L. A. Emulating a multi-arm randomized controlled trial of antihypertensive treatments for chronic kidney disease using observational data: How do the results compare with network meta-analysis? *Pharmacoepidemiology and Drug Safety*. August 2017;doi:http://dx.doi.org/10.1002/pds.4275

122. De Albeniz XGH, M.: Logan, R.: Price, M.: Hsu, J. Continuation of annual screening mammograms and breast-cancer mortality in women over 70. *Annals of Oncology*. October 2019;doi:http://dx.doi.org/10.1093/annonc/mdz263.016

123. De Albeniz XGH, J.: Hernan, M. A. Causal estimands in survival analysis with competing events: An application to breast cancer screening. *Pharmacoepidemiology and Drug Safety*. October 2020;doi:https://dx.doi.org/10.1002/pds.5114

124. Deakin C, De Stavola B, Littlejohn G, et al. EMULATING A TARGET TRIAL of ADALIMUMAB VERSUS TOFACITINIB in PATIENTS with RHEUMATOID ARTHRITIS: A COMPARATIVE EFFECTIVENESS ANALYSIS USING the OPAL REAL-WORLD DATASET. Conference Abstract. *Annals*

© 2023 Hansford HJ et al. *JAMA Network Open.*

of the Rheumatic Diseases. June 2022;81(Supplement 1):624-625. doi:https://dx.doi.org/10.1136/annrheumdis-2022-eular.2515

125. Ernandez J, Kaul S, Fleishman A, et al. Adjuvant Chemotherapy Plus Radiotherapy Versus Chemotherapy Alone for Locally Advanced Bladder Cancer after Radical Cystectomy. Conference Abstract. *Journal of Urology*. May 2022;207(SUPPL 5):e489. doi:https://dx.doi.org/10.1097/JU.0000000000002574.06

126. Fu EE, M.: Carrero, J. J.: Putter, H.: Clase, C. M.: Caskey, F.: Szymczak, M.: Torino, C.: Chesnaye, N.: Jager, K. J.: Wanner, C.: Dekker, F. W.: Van Diepen, M. When to initiate dialysis to reduce mortality and cardiovascular events in advanced CKD: A nationwide cohort study. *Nephrology Dialysis Transplantation*. May 2021;doi:http://dx.doi.org/10.1093/ndt/gfab122.001

127. Gaber C, Shaheen NJ, Edwards JK, et al. Comparative effectiveness of trimodal therapy versus definitive chemoradiation in older adults with locally advanced esophageal cancer. Conference Abstract. *Journal of Clinical Oncology Conference: Annual Meeting of the American Society of Clinical Oncology, ASCO*. 2022;40(16 Supplement 1)doi:https://dx.doi.org/10.1200/JCO.2022.40.16_suppl.e16093

128. Galaznik AB, M.: Lempernesse, B.: Ransom, J.: Shilnikova, A. ABSTRACT ONLY Pmu8 a Systematic Approach for Synthetic Replication of Clinical Trial Cohorts Using Retrospective Real-World and Clinical Trial Data. *Value in Health*. 2019;doi:http://dx.doi.org/10.1016/j.jval.2019.04.1171

129. Garcia Rodriguez LAG, M. S.: Soriano, L. C. Low-dose acetylsalicylic acid and risk of colorectal cancer: Evidence from a primary care study in the UK. *Gastroenterology*. April 2015;

130. Garcia-Albeniz XH, J.: Hernan, M. A. A word of caution: All-cause mortality and residual confounding in Medicare claims data. *Pharmacoepidemiology and Drug Safety*. August 2018;doi:http://dx.doi.org/10.1002/pds.4629

131. Gehr AL, Y.: Ghabach, B.: Narra, K.: Neerukonda, L. S.: Bullock, J.: Ojha, R. P. ABSTRACT ONLY Timing of adjuvant chemotherapy initiation and colon cancer survival. *Journal of Clinical Oncology Conference*. 2019;doi:http://dx.doi.org/10.1200/JCO.2019.37.31-suppl.119

132. Granger E, Davies G, Keogh R. Investigating the effects of dornase alfa and hypertonic saline used in combination on lung function in people with cystic fibrosis. Conference Abstract. *Journal of Cystic Fibrosis*. June 2022;21(Supplement 1):S13. doi:https://dx.doi.org/10.1016/S1569-1993%2822%2900189-8

133. Granger EF, F.: Keogh, R. Estimating the long-term effects of insulin on outcomes in cystic fibrosis-related diabetes: a target trial approach. *Journal of Cystic Fibrosis*. January 2021;doi:http://dx.doi.org/10.1016/S1569-1993%2821%2901095-X

134. Haller MCW, C.: Strohmaier, S.: Kammer, M.: Heinze, G.: Oberbauer, R. Cannot retrieve FT - Survival time gained by kidney transplantation compared to remaining waitlisted on dialysis: A national registry study using target trial emulation. *Journal of the American Society of Nephrology*. 2021;

135. Harris JS, J. A. C.: Reeves, B. C.: Johnson, T. W.: Benedetto, U.: Lasserson, D.: Loke, Y.: Mumford, A.: Rogers, C. A.: Pufulete, M. ABSTRACT ONLY The ADAPTT Study: Using routinely-collected data to emulate a randomised trial. *Trials Conference: 5th International Clinical Trials Methodology Conference, ICTMC*. 2019;doi:http://dx.doi.org/10.1186/s13063-019-3688-6

136. Himmelreich JCLV, S.: Camm, A. J.: Harskamp, R. E.: Pieper, K. S.: Fox, K. A. A.: Bassand, J. P.: Fitzmaurice, D. A.: Goldhaber, S. Z.: Goto, S.: Haas, S.: Misselwitz, F.: Turpie, A. G. G.:

© 2023 Hansford HJ et al. *JAMA Network Open.*

Verheugt, F. W. A.: Kakkar, A. K. Comparing rivaroxaban and apixaban in GARFIELD-AF according to ROCKET AF and ARISTOTLE trial selection criteria. *European Heart Journal*. 2021;doi:http://dx.doi.org/10.1093/eurheartj/ehab724.0560

137. Huitfeldt A. Emulation of Target Trials to Study the Effectiveness and Safety of Medical Interventions. *Journal*. 2015;

138. Iyer GSN. Safety and Effectiveness of Extended Treatment with Oral Anticoagulants among Patients with Unprovoked Venous Thromboembolism. *Journal*. 2021;

139. Izdebski AT, Patrick: Lalisang, Robbert C. A.: McHugh, Dean M.: Entjes, Robert: van der Meer, Nardo J. M.: Dongelmans, Dave A.: Boelens, Age D.: Rigter, Sander: Hendriks, Stefaan H. A.: de Jong, Remko: Kamps, Marlijn J. A.: Peters, Marco: Karakus, Attila: Gommers, Diederik: Ramnarain, Dharmanand: Wils, Evert-Jan: Achterberg, Sefanja: Nowitzky, Ralph: van den Tempel, Walter: de Jager, Cornelis P. C.: Nooteboom, Fleur G. C. A.: Oostdijk, Evelien A. N.: Koetsier, Peter: Cornet, Alexander D.: Reidinga, Auke C.: de Ruijter, Wouter: Bosman, Rob J.: Frenzel, Tim: Urlings-Strop, Louise C.: de Jong, Paul: Smit, Ellen G. M.: Cremer, Olaf L.: van Osch, Frits H. M.: Faber, Harald J.: Lens, Judith: Brunnekreef, Gert B.: Festen-Spanjer, Barbara: Dormans, Tom: Simons, Bram: Rijkeboer, Annemieke: Dijkstra, Annemieke: Arbous, M. Sesmu: Aries, Marcel J. H.: Beukema, Menno: van Raalte, Rutger: van Tellingen, Martijn: van den Oever, Niels C. Gritters: Elbers, Paul W. G.: Cinà, Giovanni. A pragmatic approach to estimating average treatment effects from EHR data: the effect of prone positioning on mechanically ventilated COVID-19 patients. *arXiv: Learning*. 2021;doi:NA

140. Jackson JW. A general framework for diagnosing confounding of time-varying and other joint exposures. *NA*. 2015;doi:NA

141. Jackson JW. Diagnosing Covariate Balance Across Levels of Right-Censoring Before and After Application of Inverse-Probability-of-Censoring Weights. *American journal of epidemiology*. 2019;doi:10.1093/aje/kwz136

142. Jackson JW, Garcia-Albeniz, XA. Studying the effects of nonindicated medications on cancer: Etiologic versus action-focused analysis of epidemiologic data. *Cancer epidemiology, biomarkers & prevention : a publication of the American Association for Cancer Research, cosponsored by the American Society of Preventive Oncology*. 2018;doi:10.1158/1055-9965.epi-17-0862

143. Keogh RHG, Jon Michael: Seaman, Shaun R.: Davies, Gwyneth: Vansteelandt, Stijn. Causal inference in survival analysis using longitudinal observational data: Sequential trials and marginal structural models. *Journal*. 2021;

144. Keogh RHS, S.: Gran, J.: Vansteelandt, S.: Bilton, D.: Carr, S. B. Emulating a target trial using the UK cystic fibrosis registry: The causal effect of DNASE on 5-year survival. *Pediatric Pulmonology*. 2019;doi:http://dx.doi.org/10.1002/ppul.22495

145. Keshet AR, Hagai: Shilo, Smadar: Barbash-Hazan, Shiri: Amit, Guy: Bivas-Benita, Maytal: Yanover, Chen: Girshovitz, Irena: Akiva, Pinchas: Ben-Haroush, Avi: Hadar, Eran: Wiznitzer, Arnon: Segal, Eran. Estimating the effect of cesarean delivery on long term childhood health across two countries. *medRxiv*. 2020;doi:NA

146. Kilkenny M. Target Trial Approach in Comparative Effectiveness Studies for Stroke. Conference Abstract. *Neuroepidemiology*. 2022;56(SUPPL 1):42.

147. Kraglund F, Christensen DH, Jepsen P. Statins, but not aspirin, reduce the risk of hepatocellular carcinoma and mortality in Danish patients with cirrhosis due to alcohol-related

© 2023 Hansford HJ et al. *JAMA Network Open.*

liver disease: a nationwide causal study. Conference Abstract. *Journal of Hepatology*. July 2022;77(Supplement 1):S172-S173. doi:https://dx.doi.org/10.1016/S0168-8278%2822%2900724-3

148. Kuehne FA, M.: Hess, L. M.: Faries, D. E.: Matteucci Gothe, R.: Gothe, H.: Beyrer, J.: Zeimet, A. G.: Stojkov, I.: Muhlberger, N.: Oberaigner, W.: Marth, C.: Siebert, U. ABSTRACT ONLY
Assessing type and impact of biases potentially occurring when analyzing real world evidence: The case of second line treatment for ovarian cancer. *Value in Health*. 2018;

149. Kuehne FR, U.: Paracha, N.: Yeh, J. M.: Sabate, E.: Siebert, U. Estimating Treatment-Switching Bias in a Randomized Clinical Trial of Ovarian Cancer Treatment: Combining Causal Inference with Decision-Analytic Modeling. *Medical decision making : an international journal of the Society for Medical Decision Making*. 2022;doi:https://dx.doi.org/10.1177/0272989X211026288

150. Larochelle MR, Lodi S, Yan S, Clothier BA, Goldsmith ES, Bohnert ASB. Comparative Effectiveness of Opioid Tapering or Abrupt Discontinuation vs No Dosage Change for Opioid Overdose or Suicide for Patients Receiving Stable Long-term Opioid Therapy. *JAMA Network Open*. 12 Aug 2022;5(8):E2226523. doi:https://dx.doi.org/10.1001/jamanetworkopen.2022.26523

151. Lindbohm JVM, Nina: Sipilä, Pyry N.: Singh-Manoux, Archana: Runz, Heiko: Livingston, Gill: Seshadri, Sudha: Xavier, Ramnik: Hingorani, Aroon D.: Ripatti, Samuli: Kivimäki, Mika. Immune system and blood-brain barrier-wide biomarker analyses provide causal evidence for autoimmunity in dementia. *NA*. 2022;doi:10.1101/2022.02.17.22271136

152. Lindquist A, Hastie R, Kennedy A, et al. Childhood developmental vulnerability following elective birth at 39 weeks'. Conference Abstract. *BJOG: An International Journal of Obstetrics and Gynaecology*. June 2022;129(Supplement 1):74. doi:https://dx.doi.org/10.1111/1471-0528.10_17178

153. Madenci A. Causal Inference in Health Services Research. *Journal*. 2020 2020;

154. Mahévas MT, Viet-Thi: Roumier, Mathilde: Chabrol, Amélie: Paule, Romain: Guillaud, Constance: Gallien, S.: Lepeule, Raphaël: Szwebel, Tali-Anne: Lescure, Xavier: Schlemmer, Frédéric: Matignon, Marie: Khellaf, Medhi: Crickx, Etienne: Terrier, Benjamin: Morbieu, Caroline: Legendre, Paul: Dang, Julien: Schoindre, Yoland: Pawlotski, Jean-Michel: Michel, Marc: Perrodeau, Elodie: Carlier, Nicolas: Roche, Nicolas: de Lastours, Victoire: Mouthon, Luc: Audureau, Etienne: Ravaud, Philippe: Godeau, Bertrand: Costedoat-Chalumeau, Nathalie. No evidence of clinical efficacy of hydroxychloroquine in patients hospitalized for COVID-19 infection with oxygen requirement: results of a study using routinely collected data to emulate a target trial. *NA*. 2020;doi:10.1101/2020.04.10.20060699

155. Maringe CBM, S.: Exarchakou, A.: Smith, M.: Rachet, B.: Belot, A.: Leyrat, C. Reflection on modern methods: Trial emulation in the presence of immortal-time bias. Assessing the benefit of major surgery for elderly lung cancer patients using observational data. *International Journal of Epidemiology*. 1-Oct 2020;doi:https://dx.doi.org/10.1093/ije/dyaa057

156. Martinez-Sanz J, Ron R, Moreno E, et al. 2DR VS 3DR INTEGRASE INHIBITOR-BASED REGIMENS INITIATION and CD4+/CD8+ RATIO RECOVERY. Conference Abstract. *Topics in Antiviral Medicine*. March 2022;30(1 SUPPL):184.

© 2023 Hansford HJ et al. *JAMA Network Open.*

157. Matthews AD, Issa J.: Fröbert, Ole: Lindahl, Bertil: James, Stefan: Feychting, Maria: Jernberg, Tomas: Berglund, Anita: Hernán, Miguel A. Extending treatment effects from a randomized trial using observational data: an application to coronary thrombus aspiration. *NA*. 2021;doi:10.1101/2021.09.06.21263156
158. Matulonis U, Mirza M, Malinowska I, Perhanidis J, Woodward T, Kalilani L. Real-world clinical outcomes with poly (adenosine diphosphate [ADP]-ribose) polymerase inhibitors as second-line maintenance therapy in patients with recurrent ovarian cancer in the United States (353). Conference Abstract. *Gynecologic Oncology*. August 2022;166(Supplement 1):S180-S181. doi:https://dx.doi.org/10.1016/S0090-8258%2822%2901575-X
159. McConeghy KWB, B.: Huang, A. W.: White, E. M.: Feifer, R. A.: Blackman, C.: Santostefano, C. M.: Lee, Y.: DeVone, F.: Halladay, C. W.: Rudolph, J. L.: Zullo, A. R.: Mor, V.: Gravenstein, S. Effectiveness of a SARS-CoV-2 mRNA vaccine booster dose for prevention of infection, hospitalization or death in two nation-wide nursing home systems. *medRxiv*. 2022;doi:https://dx.doi.org/10.1101/2022.01.25.22269843
160. McDermott GF, X.: Cook, C.: Stone, J.: Zhang, Y.: Wallace, Z. Post-induction anca titer does not predict mortality or renal outcomes: A target trial emulation study. *Arthritis and Rheumatology*. September 2021;doi:https://dx.doi.org/10.1002/art.41966
161. McGinty EE, Tormohlen KN, Barry CL, Bicket MC, Rutkow L, Stuart EA. Protocol: mixed-methods study of how implementation of US state medical cannabis laws affects treatment of chronic non-cancer pain and adverse opioid outcomes. *Implementation Science*. 2021;16(1):1-13.
162. Mei H. Clinical Treatment Human Disease Networks and Comparative Effectiveness Research: Analyses of the Medicare Administrative Data. *Journal*. 2021 2021;
163. Miranda RS, Tinne: De Schreye, Robrecht: Faes, Kristof: Van Den Noortgate, Nele: Cohen, Joachim: Van den Block, Lieve. Improved quality of care and reduced healthcare costs at the end-of-life among older people with dementia who received palliative home care: A nationwide propensity score-matched decedent cohort study. *Palliative medicine*. 2021;doi:10.1177/02692163211019321
164. Moreno-Betancur MM, Paul: Becker, Denise: Patton, George C.: Carlin, John B. Defining mediation effects for multiple mediators using the concept of the target randomized trial. *arXiv: Methodology*. 2019;doi:NA
165. Moura LM, Westover, M. Brandon, Kwasnik, David, Cole, Andrew J. and Hsu, John. Causal inference as an emerging statistical approach in neurology: an example for epilepsy in the elderly. *Clinical epidemiology*. 2016;doi:10.2147/CLEP.S121023
166. Narayan V. Comparing Cardiovascular Outcomes with Degarelix or Leuprolide as Prostate Cancer Therapy - Applying Real-world Data to Clinical Trial Emulation. *JAMA Network Open*. 22-Oct 2021;doi:http://dx.doi.org/10.1001/jamanetworkopen.2021.31429
167. Nguyen VG, Yavlinsky A, Beale S, et al. Comparative effectiveness of different primary vaccination courses on mRNA based booster vaccines against SARs-COV-2 infections: A time-varying cohort analysis using trial emulation in the Virus Watch community cohort. *medRxiv*. 2022:2022.02.04.22270479. doi:10.1101/2022.02.04.22270479
168. Nguyen VGY, A.: Beale, S.: Hoskins, S.: Lampos, V.: Braithwaite, I.: Byrne, T. E.: Fong, W. L. E.: Fragaszy, E.: Geismar, C.: Kovar, J.: Navaratnam, A. M. D.: Patel, P.: Shrotri, M.: Weber, S.: Hayward, A. C.: Aldridge, R. W.: Michie, S.: Hardelid, P.: Wijlaars, L.: Nastouli, E.: Spyer, M.:

© 2023 Hansford HJ et al. *JAMA Network Open.*

Killingley, B.: Cox, I.: McKendry, R. A.: Cheng, T.: Liu, Y.: Gibbs, J.: Gilson, R.: Lopez Bernal, J.: Johnson, A. M.: Rodger, A. Comparative effectiveness of ChAdOx1 versus BNT162b2 vaccines against SARS-CoV-2 infections in England and Wales: A cohort analysis using trial emulation in the Virus Watch community data. *medRxiv*. 2021;doi:https://dx.doi.org/10.1101/2021.12.21.21268214

169. Nielsen PB, Søgaard M, Skjøth F, Larsen TB, Lip GYH, Hernán M. IMPORTANCE OF THE ANALYTIC APPROACH WHEN COMPARING THE EFFECTIVENESS OF TREATMENTS: AN APPLICATION TO REDUCED DOSE NOACS FOR STROKE PREVENTION IN ATRIAL FIBRILLATION. *Journal of the American College of Cardiology*. 2020;75(11_Supplement_1):364-364. doi:doi:10.1016/S0735-1097(20)30991-8

170. Parra CO, Waernbaum I, Schön S, Goetghebeur E. Trial emulation and survival analysis for disease incidence registers: a case study on the causal effect of pre-emptive kidney transplantation. *arXiv preprint arXiv:201111771*. 2020;

171. Piske MT, T.: Krebs, E.: Hongdilokkul, N.: Bruneau, J.: Greenland, S.: Gustafson, P.: Karim, M. E.: McCandless, L. C.: MacLure, M.: Platt, R. W.: Siebert, U.: Eugenia Socias, M.: Tsui, J. I.: Wood, E.: Nosyk, B. Comparative effectiveness of buprenorphine-naloxone versus methadone for treatment of opioid use disorder: A population-based observational study protocol in British Columbia, Canada. *BMJ Open*. 9-Sep 2020;doi:http://dx.doi.org/10.1136/bmjopen-2019-036102

172. Prats-Uribe A, Berencsi K, Carr A, et al. The performance of preference-based instrumental variables to emulate a randomized clinical trial of comparative medical device effectiveness. Wiley; 2019:431-432.

173. Prats-Uribe AA, M. S.: Strauss, V. Y.: Prieto-Alhambra, D. The performance of instrumental variables in medical device epidemiology: A simulation study of non-normally distributed continuous outcome data based on real world post-operative PROMs. *Pharmacoepidemiology and Drug Safety*. October 2020;doi:https://dx.doi.org/10.1002/pds.5114

174. Puig IS, M.: Nyssen, O. P.: Fiorini, G.: Vaira, D.: Saracino, I.: Perez-Aisa, A.: Fernandez-Moreno, N.: Santaella, I.: Castro-Fernandez, M.: Bujanda, L.: Lucendo, A.: Areia, M.: Gasbarrini, A.: Romano, M.: Gravina, A.: Marcos-Pinto, R.: Megraud, F.: O'Morain, C.: Gisbert, J. P. Real-world comparative effects of three-in-one single capsule bismuth quadruple therapy vs non-bismuth quadruple concomitant therapy: Interim analysis of the European Registry on H. pylori Management (Hp-EuReg). *Helicobacter Conference: 33rd International Workshop on Helicobacter and Microbiota in Inflammation and Cancer Virtual*. 2020;doi:https://dx.doi.org/10.1111/hel.12745

175. Reeves BCS, L. J.: Taylor, J.: Hogg, R.: Rogers, C. A.: Wordsworth, S.: Townsend, D.: Muldrew, A.: Peto, T.: Violato, M.: Dakin, H.: Cappel-Porter, H.: Mills, N.: O'Reilly, D.: Harding, S. P.: Chakravarthy, U. The Effectiveness, cost-effectiveness and acceptability of Community versus Hospital Eye Service follow-up for patients with neovascular age-related macular degeneration with quiescent disease (ECHoES): A virtual randomised balanced incomplete block trial. *Health Technology Assessment*. November 2016;doi:http://dx.doi.org/10.3310/hta20800

176. Rentsch CTB, J. A.: Tomlinson, L.: Gellad, W. F.: Alcorn, C.: Kidwai-Khan, F.: Skanderson, M.: Brittain, E.: King, J. T.: Ho, Y. L.: Eden, S.: Kundu, S.: Lann, M. F.: Greevy, R. A.: Ho, P. M.: Heidenreich, P. A.: Jacobson, D. A.: Douglas, I. J.: Tate, J. P.: Evans, S. J. W.: Atkins, D.: Justice, A.

© 2023 Hansford HJ et al. *JAMA Network Open.*

C.: Freiberg, M. S. Safety and effectiveness of prophylactic anticoagulation for prevention of COVID-19 mortality: A nationwide cohort study of hospitalised patients in the US. *Pharmacoepidemiology and Drug Safety*. August 2021;doi:http://dx.doi.org/10.1002/pds.5305

177. Rodriguez CA. Causal Inference for the Treatment of Multidrug-Resistant Tuberculosis. *Journal*. 2021 2021;

178. Rossides M. Epidemiological Aspects of Sarcoidosis: Risk Factors and Long-Term Consequences. *Journal*. 2021 2021;

179. Sabathe CC, R.: Vukusic, S.: Leray, E.: Mathey, G.: De Seze, J.: Ciron, J.: Wiertlewski, S.: Ruet, A.: Pelletier, J.: Zephir, H.: Michel, L.: Lebrun-Frenay, C.: Moisset, X.: Thouvenot, E.: Camdessanche, J. P.: Bakchine, S.: Stankoff, B.: Al Khedr, A.: Cabre, P.: Maillart, E.: Berger, E.: Heinzlef, O.: Hankiewicz, K.: Moreau, T.: Gout, O.: Bourre, B.: Wahab, A.: Labauge, P.: Montcuquet, A.: Defer, G.: Maurousset, A.: Maubeuge, N.: Boulos Dalia, D.: Ben Nasr, H.: Nifle, C.: Casez, O.: Laplaud, D. A.: Foucher, Y. Improving the decision to switch from first to second-line therapy in MS: A dynamic scoring system. *Multiple Sclerosis Journal*. October 2021;doi:http://dx.doi.org/10.1177/13524585211044647

180. Sanchez AR, V. H.: Navarro, M.: Pineda, M. P.: Caballol Oliva, B.: Moreno, L.: Carballal, S.: Rodriguez-Alonso, L.: Teresa, R. Y. C.: Llort, G.: Romero, C.: Pinol, V.: Lopez, A.: Salces, I.: Pico Sala, M. D.: Rivas, L.: Bujanda Fernandez de Pierola, L.: Garzon, M.: Pizarro Moreno, E.: Martinez De Castro, E.: Lopez-Arias, M. J.: Poves, C.: Garau Colom, C.: Rodriguez-Alcalde, D.: Herraiz, M.: Alvarez Urturi, C.: Bessa, X.: Dacal Rivas, A.: Carrillo-Palau, M.: Cid-Gomez, L.: Ponce, M.: Suarez Gonzalez, A.: Saperas Franch, E.: Aguirre, E.: Morales-Romero, B.: Ocana, T.: Rivero Sanchez, L.: Jung, G.: Cubiella, J.: Jover, R.: Rodriguez-Moranta, F.: Balmana, J.: Castells, A.: Capella, G.: Serra-Burriel, M.: Moreira Ruiz, L.: Pellise Urquiza, M.: Balaguer, F. Association between colonoscopy quality indicators, diagnostic yield, and post colonoscopy colorectal cancer incidence in lynch syndrome. *United European Gastroenterology Journal*. October 2020;doi:https://dx.doi.org/10.1177/2050640620927344

181. Sankaran SKP, Sonis S. Relationships and Impact of Ulcerative Mucositis on Patients Hospitalized for Cancer Therapy: Real-World Evidence Grounded on Big Data. Conference Abstract. *Supportive Care in Cancer*. June 2022;30(Supplement 1):S65. doi:https://dx.doi.org/10.1007/s00520-022-07099-y

182. Schaufler C, Kaul S, Fleishman A, et al. Immediate Radiotherapy Versus Observation in Patients with Node-Positive Prostate Cancer after Radical Prostatectomy. Conference Abstract. *Journal of Urology*. May 2022;207(SUPPL 5):e403-e404. doi:https://dx.doi.org/10.1097/JU.0000000000002564.04

183. Schmidt MS, H. T.: Pedersen, L. Cardiovascular risks associated with diclofenac initiation: A nationwide emulated randomized trial. *European Heart Journal*. August 2017;doi:http://dx.doi.org/10.1093/eurheartj/ehx502.1015

184. Sharma AO, Gina: Holodniy, Mark. Effectiveness of a third dose of BNT162b2 or mRNA-1273 vaccine for preventing post-vaccination COVID-19 infection: an observational study. *NA*. 2021;doi:10.1101/2021.11.29.21266777

185. Siebert UK, F.: Arvandi, M.: Hess, L. M.: Faries, D. E.: Beyrer, J.: Matteucci Gothe, R.: Gothe, H.: Stojkov, I.: Rochau, U.: Oberaigner, W.: Zeimet, A. G.: Marth, C. Comparative effectiveness analysis using real world evidence (RWE) and applying a causal marginal structural

© 2023 Hansford HJ et al. *JAMA Network Open.*

model: The case of second-line treatment in patients with ovarian cancer. *Value in Health*. May 2018;
186. Smith LH. *Bias bounds and target trials for causal inference in observational epidemiology*. Harvard University; 2021.
187. Soto-Becerra PC, Carlos: Hurtado-Roca, Yamilee: Araujo-Castillo, Roger V. Real-World Effectiveness of hydroxychloroquine, azithromycin, and ivermectin among hospitalized COVID-19 patients: Results of a target trial emulation using observational data from a nationwide Healthcare System in Peru. *SSRN Electronic Journal*. 2020;doi:10.2139/ssrn.3710623
188. Steen J, Vansteelandt S, Decruyenaere J, Van Biesen W. Target Trial Emulation on Timing of Start of Renal Replacement Therapy in Acute Kidney Injury: Lessons Learned. Conference Abstract. *Nephrology Dialysis Transplantation*. May 2022;37(SUPPL 3):i266-i268. doi:https://dx.doi.org/10.1093/ndt/gfac135.018
189. Subramaniam H. The Effect of Antiretroviral Therapy on Preterm Birth in a U.S Population of Women Living with HIV: A Reexamination of Analysis Methods. *Journal*. 2020 2020;
190. Sun JY, J.: Sarvet, A.: Lin, P. I. D.: Toh, S.: Block, J. Risk of type 2 diabetes among children initiating anticonvulsant mood stabilizers: Application of a target trial approach. *Pharmacoepidemiology and Drug Safety*. August 2021;doi:http://dx.doi.org/10.1002/pds.5305
191. Thomas DSL, Aaron Y.: Müller, Philipp L.: Schwartz, Roy: Olvera-Barrios, Abraham: Warwick, Alasdair: Patel, Praveen J.: Heeren, Tjebo F. C.: Egan, Catherine A.: Taylor, Paul: Tufail, Adnan. Contextualising Single-Arm Ophthalmology Trials With Real-World Data: An Emulated Target Trial Comparing The Efficacy of Interventions For Neovascular Age-Related Macular Degeneration. *NA*. 2020;doi:10.1101/2020.08.10.20171538
192. Tran V-TM, Matthieu: Sadr, Firouze Bani: Robineau, Olivier: Perpoint, Thomas: Perrodeau, Elodie: Gallay, Laure: Ravaud, Philippe: Goehringer, François: Lescure, Xavier: Cocorico, N. A. Association between corticosteroids and intubation or death among patients with COVID-19 pneumonia in non-ICU settings: an observational study using of real-world data from 51 hospitals in France and Luxembourg. *NA*. 2020;doi:10.1101/2020.09.16.20195750
193. Trevisan M. *Drug Safety and Effectiveness in Relation to Kidney Function: A Pharmacoepidemiological Approach*. Karolinska Institutet (Sweden); 2021.
194. Ughetto AE, J.: David, H.: Nagot, N.: Bazalgette, F.: Marin, G.: Mourad, M.: Kollen, S.: Gaudard, P.: Colson, P. Impact of Temporary Mechanical Circulatory Support on mortality in Cardiogenic Shock: An emulated target trial with a prospective, multicenter, French cohort study. *Intensive Care Medicine Experimental Conference: European Society of Intensive Care Medicine Annual Congress, ESICM*. 2021;doi:http://dx.doi.org/10.1186/s40635-021-00413-8
195. Urner MB, A. G.: Li Bassi, G.: Brodie, D.: Dalton, H. J.: Ferguson, N. D.: Heinsar, S.: Hodgson, C.: Peek, G.: Shekar, K.: Suen, J. Y.: Fraser, J.: Fan, E. Venovenous Extracorporeal Membrane Oxygenation in Patients with Acute Respiratory Failure from COVID-19. *Intensive Care Medicine Experimental Conference: European Society of Intensive Care Medicine Annual Congress, ESICM*. 2021;doi:http://dx.doi.org/10.1186/s40635-021-00413-8
196. Van Santen DKB, A.: Master, A.: Lodi, S.: Prins, M. Harm reduction revisited: The causal effect of the Dutch approach towards people who inject drugs on HIV, hepatitis B and C infection risk. *Journal of the International AIDS Society Conference: 23rd International AIDS Conference Virtual*. 2020;doi:https://dx.doi.org/10.1002/jia2.25547

© 2023 Hansford HJ et al. *JAMA Network Open.*

197. Wallisch C, Strohmaier S, Kammer M, et al. Survival Benefit of Kidney Transplantation Compared to Long-Term Dialysis Across Ages: A Retrospective Cohort Study Using Target Trial Emulation. *Available at SSRN 3991625*. 2021;

198. Watson K. Epidemiologic Approaches to Investigating the Relationship between Insulin Resistance and Major Depressive Disorder. *Journal*. 2020 2020;

199. Webb BJB, Whitney R.: Vento, Todd J.: Butler, Allison M.: Grisel, Nancy: Brown, Samuel M.: Peltan, Ithan D.: Spivak, Emily S.: Shah, Mark B.: Sakata, Theadora: Wallin, Anthony: Stenehjem, Eddie: Poulsen, Greg: Bledsoe, Joseph. Real-World Effectiveness and Tolerability of Monoclonal Antibodies for Ambulatory Patients with Early COVID-19. *NA*. 2021;doi:10.1101/2021.03.15.21253646

200. Wintzell V. Pharmacoepidemiologic Studies of Drug Safety in Pediatric Chronic Inflammatory Disease. *Journal*. 2021 2021;

201. Zang CZ, Hao: Xu, Jie: Zhang, Hansi: Fouladvand, Sajjad: Havaldar, Shreyas: Cheng, Feixiong: Chen, Kun: Chen, Yong: Glicksberg, Benjamin S.: Chen, Jin: Bian, Jiang: Wang, Fei. High-Throughput Clinical Trial Emulation with Real World Data and Machine Learning: A Case Study of Drug Repurposing for Alzheimer's Disease. *NA*. 2022;doi:10.1101/2022.01.31.22270132

202. Zhao J, Sjolander A, Edgren G. Effect of blood donor sex and parity on mortality among adult patients transfused with red-cells: A nationwide natural experiment. Conference Abstract. *Vox Sanguinis*. 2022;117(SUPPL 1):50. doi:https://dx.doi.org/10.1111/vox.13285

Other (40)

203. Abstracts from the 2020 Annual Meeting of the Society of General Internal Medicine. *Journal of General Internal Medicine*. 2020/07/01 2020;35(1):1-779. doi:10.1007/s11606-020-05890-3

204. Angriman F, Rosella LC, Lawler PR, et al. Renin-Angiotensin System Inhibitors and Major Cardiovascular Events after Sepsis. *Ann Am Thorac Soc*. Oct 17 2022;doi:10.1513/AnnalsATS.202207-615OC

205. Ben-Michael E, Feller A, Stuart EA. A Trial Emulation Approach for Policy Evaluations with Group-level Longitudinal Data. *Epidemiology*. 2021;32(4):533-540. doi:10.1097/ede.0000000000001369

206. Buchanan ALB, S.: Goedel, William C.: King, Maximilian: Murray, Eleanor J.: Friedman, Samuel R.: Halloran, M. Elizabeth: Marshall, Brandon D. L. Disseminated Effects in Agent-Based Models: A Potential Outcomes Framework and Application to Inform Preexposure Prophylaxis Coverage Levels for HIV Prevention. *American journal of epidemiology*. 2020;doi:10.1093/aje/kwaa239

207. Buranupakor TPT, Jayanton Patumanond, Phichayut Phinyo. Emulation of a Target Trial to Evaluate the Causal Effect of Palliative Care Consultation on the Survival Time of Patients with Hepatocellular Carcinoma. *Cancers*. 2021: 2021-03-05 2021;doi:http://dx.doi.org/10.3390/cancers13050992

208. Chemaitelly H, AlMukdad S, Ayoub HH, et al. Effectiveness of the BNT162b2 vaccine against SARS-CoV-2 infection among children and adolescents in Qatar. *NA*. 2022;NA(NA):NA-NA. doi:10.1101/2022.07.26.22278045

209. Chemaitelly H, Ayoub HH, AlMukdad S, et al. Protection of prior natural infection compared to mRNA vaccination against SARS-CoV-2 infection and severe COVID-19 in Qatar. *NA*. 2022;NA(NA):NA-NA. doi:10.1101/2022.03.17.22272529

210. Chemaitelly H, Ayoub HH, Tang P, et al. Immune protection against SARS-CoV-2 re-reinfection and immune imprinting. Preprint. *medRxiv*. 2022;24doi:https://dx.doi.org/10.1101/2022.08.23.22279026

211. Chemaitelly H, Nagelkerke N, Ayoub HH, et al. Duration of immune protection of SARS-CoV-2 natural infection against reinfection in Qatar. *NA*. 2022;NA(NA):NA-NA. doi:10.1101/2022.07.06.22277306

212. Chen X. Causal Inference and Prediction on Observational Data with Survival Outcomes. 2020;

213. Chen Z, Zhang H, George TJ, et al. Simulating Colorectal Cancer Trials Using Real-World Data. *JCO clinical cancer informatics*. 2022;6(6):e2100195-NA. doi:10.1200/cci.21.00195

214. Dryden-Peterson S, Kim A, Kim AY, et al. Nirmatrelvir plus ritonavir for early COVID-19 and hospitalization in a large US health system. Preprint. *medRxiv*. 2022;16doi:https://dx.doi.org/10.1101/2022.06.14.22276393

215. Heil TC, Verdaasdonk EGG, Maas HAAM, et al. Improved Postoperative Outcomes after Prehabilitation for Colorectal Cancer Surgery in Older Patients: An Emulated Target Trial. *Annals of surgical oncology*. 2022;05doi:https://dx.doi.org/10.1245/s10434-022-12623-9

216. Hoffman KL, Schenck EJ, Satlin MJ, et al. Comparison of a Target Trial Emulation Framework vs Cox Regression to Estimate the Association of Corticosteroids With COVID-19 Mortality. *JAMA Netw Open*. Oct 3 2022;5(10):e2234425. doi:10.1001/jamanetworkopen.2022.34425

217. Hoffman KL, Schenck EJ, Satlin MJ, et al. Corticosteroids in COVID-19: Optimizing Observational Research through Target Trial Emulations. *medRxiv*. Jun 7 2022;doi:10.1101/2022.05.27.22275037

218. Hoffman KL, Schenck EJ, Satlin MJ, et al. Comparison of a Target Trial Emulation Framework vs Cox Regression to Estimate the Association of Corticosteroids With COVID-19 Mortality. *JAMA network open*. 03 Oct 2022;5(10):e2234425. doi:https://dx.doi.org/10.1001/jamanetworkopen.2022.34425

219. Hoffman KL, Schenck EJ, Satlin MJ, et al. Corticosteroids in COVID-19: Optimizing Observational Research through Target Trial Emulations. *medRxiv : the preprint server for health sciences*. 2022;NA(NA):NA-NA. doi:10.1101/2022.05.27.22275037

220. Huitfeldt A, Kalager M, Robins JM, Hoff G, Hernán MA. Methods to estimate the comparative effectiveness of clinical strategies that administer the same intervention at different times. *Current epidemiology reports*. 2015;2(3):149-161.

221. Ioannou GN, Bohnert ASB, O'Hare AM, et al. Effectiveness of mRNA COVID-19 vaccine boosters against infection, hospitalization and death: A target trial emulation in the omicron (B.1.1.529) variant era. Preprint. *medRxiv*. 2022;16doi:https://dx.doi.org/10.1101/2022.06.15.22276466

222. Jiao T, Filion KB, Douros A, Platt RW. Antihypertensive treatment patterns in the United Kingdom. WILEY 111 RIVER ST, HOBOKEN 07030-5774, NJ USA; 2020:148-148.

© 2023 Hansford HJ et al. *JAMA Network Open.*

223. Jiao T, Platt RW, Douros A, Filion KB. Target blood pressure levels and the risk of major adverse cardiovascular events among patients with hypertension at low cardiovascular disease risk. WILEY 111 RIVER ST, HOBOKEN 07030-5774, NJ USA; 2021:350-350.
224. Kainz A, Kammer M, Reindl-Schwaighofer R, et al. EFFECT OF WAITING TIME ON MORTALITY AFTER SECOND KIDNEY TRANSPLANTATION. WILEY 111 RIVER ST, HOBOKEN 07030-5774, NJ USA; 2021:32-32.
225. Kainz A, Kammer M, Reindl-Schwaighofer R, et al. Waiting Time for Second Kidney Transplantation and Mortality. *Clin J Am Soc Nephrol*. Jan 2022;17(1):90-97. doi:10.2215/cjn.07620621
226. Kennedy J, Parker M, Seaborne M, et al. Health care use attributable to COVID-19: A propensity matched national electronic health records cohort study of 249,390 people in Wales, UK. *NA*. 2022;NA(NA):NA-NA. doi:10.1101/2022.04.21.22274152
227. Koretz RL. In US veterans, PPIs were linked to increased all-cause and some cause-specific mortality compared with H2-blockers. *Annals of internal medicine*. 2019;171(6):JC35.
228. Martire LR. Sickness absence among patients with chronic pain in Swedish specialist healthcare. 2021;
229. Nguyen VG, Yavlinsky A, Beale S, et al. Comparative effectiveness of different primary vaccination courses on mRNA based booster vaccines against SARs-COV-2 infections: A time-varying cohort analysis using trial emulation in the Virus Watch community cohort. Preprint. *medRxiv*. 2022;06doi:https://dx.doi.org/10.1101/2022.02.04.22270479
230. Nguyen VG, Zylbergsztejn A, Harron K, et al. SEN support from the start of school and its impact on unplanned hospital utilisation in children with cleft lip and palate: a demonstration target trial emulation protocol using ECHILD. Preprint. *medRxiv*. 2022;05doi:https://dx.doi.org/10.1101/2022.04.01.22273280
231. Rauh-Hain JA, Zubizarreta J, Nitecki R, et al. Survival outcomes following pregnancy or assisted reproductive technologies after breast cancer: A population-based study. *Cancer*. 2022;128(17):3243-3253. doi:10.1002/cncr.34371
232. Schnitzer ME, Guerra SF, Longo C, Blais L, Platt RW. A potential outcomes approach to defining and estimating gestational age-specific exposure effects during pregnancy. *Statistical methods in medical research*. 2022;31(2):300-314. doi:10.1177/09622802211065158
233. Simmonds KP, Burke J, Kozlowski AJ, Andary M, Luo Z, Reeves MJ. Emulating 3 Clinical Trials That Compare Stroke Rehabilitation at Inpatient Rehabilitation Facilities With Skilled Nursing Facilities. *Archives of physical medicine and rehabilitation*. 2022;103(7):1311-1319. doi:10.1016/j.apmr.2021.12.029
234. Simmonds KP, Burke J, Kozlowski AJ, Andary M, Luo ZH, Reeves MJ. Emulating 3 Clinical Trials That Compare Stroke Rehabilitation at Inpatient Rehabilitation Facilities With Skilled Nursing Facilities. Article. *Archives of Physical Medicine and Rehabilitation*. Jul 2022;103(7):1311-1319. doi:10.1016/j.apmr.2021.12.029
235. Sun JW, Young JG, Sarvet AL, et al. Comparison of Rates of Type 2 Diabetes in Adults and Children Treated With Anticonvulsant Mood Stabilizers. *JAMA network open*. 2022;5(4):e226484-e226484. doi:10.1001/jamanetworkopen.2022.6484
236. Thomas J, Manser D, Thomas P, et al. Stroke Society of Australasia Annual Scientific Meeting 13-15 October 2021, Perth Australia. *Int J Stroke*. 2021:3-34.

© 2023 Hansford HJ et al. *JAMA Network Open.*

237. Toh S, Manson JE. An analytic framework for aligning observational and randomized trial data: application to postmenopausal hormone therapy and coronary heart disease. *Statistics in biosciences*. 2013;5(2):344-360.

238. Tsuzuki S, Hayakawa K, Uemura Y, et al. Effectiveness of remdesivir in hospitalized nonsevere patients with COVID-19 in Japan: A large observational study using the COVID-19 Registry Japan. *International journal of infectious diseases : IJID : official publication of the International Society for Infectious Diseases*. 2022;118(NA):119-125. doi:10.1016/j.ijid.2022.02.039

239. Weinstein SMC, Laura C.: Helliwell, Philip S.: Ogdie, Alexis: Stephens-Shields, Alisa J. Simulation-based design of pragmatic trials in psoriatic arthritis using propensity scores. *Clinical trials (London, England)*. 2021;doi:10.1177/17407745211023840

240. Westerberg ML, R.: Holmberg, L.: Stattin, P.: Garmo, H. Simulation model of disease incidence driven by diagnostic activity. *Statistics in Medicine*. 28-Feb 2021;doi:http://dx.doi.org/10.1002/sim.8833

241. Yland JJ, Chiu YH, Rinaudo P, Hsu J, Hernan MA, Hernandez-Diaz S. Emulating a target trial of the comparative effectiveness of clomiphene citrate and letrozole for ovulation induction. *Human Reproduction*. 01 Apr 2022;37(4):793-805. doi:https://dx.doi.org/10.1093/humrep/deac005

242. Zhu J, Zhang H, Brazier E, et al. The impact of WHO's Treat All guideline on disease progression among people enrolled in HIV care in Central Africa: an observational cohort data by target trial design with multistate modeling. Preprint. *medRxiv*. 2022;30doi:https://dx.doi.org/10.1101/2022.08.27.22279144

© 2023 Hansford HJ et al. *JAMA Network Open.*

Appendix 7. Subgroup Analysis of Reporting of Target Trial Protocol and How It Was Emulated, by Reporting Guideline Use

	Value	Count (N (%))	
	Used reporting guideline?	No (n=157)	Yes (n=43)
How the protocol of the emulated target trial was reported	Not fully described	54 (34)	15 (35)
	Only in text	50 (32)	19 (44)
	Table	54 (34)	9 (21)
Both target trial protocol and its emulation described explicitly as such?	Yes	73 (46)	13 (30)
Description of how the target trial was emulated[a]			
Eligibility criteria	Yes	151 (96)	42 (98)
Treatment strategies	Yes	149 (96)	42 (98)
Assignment procedures	Yes	142 (90)	31 (72)
Outcome(s)	Yes	152 (97)	42 (98)
Follow-up	Yes	146 (93)	40 (93)
Causal contrast(s)	Yes	112 (71)	34 (79)
Analysis plan	Yes	153 (97)	41 (95)
Specification of time zero (i.e., baseline)	Yes	128 (82)	37 (86)

[a.] Operational definitions of target trial protocol items are described in Appendix 5.

© 2023 Hansford HJ et al. *JAMA Network Open.*
