# Supplementary material for: Reporting of Observational Studies Explicitly Aiming to Emulate Randomized Trials: A Systematic Review
Source: JAMA Netw Open. 2023 Sep 27;6(9):e2336023. doi: 10.1001/jamanetworkopen.2023.36023 (PMC10534275; doi:10.1001/jamanetworkopen.2023.36023)
Supplement: Supplement 2. — Data Sharing Statement [file jamanetwopen-e2336023-s002.pdf]

Data Sharing Statement

Hansford. Reporting of Observational Studies Explicitly Emulating Randomized Trials. *JAMA Netw Open*. Published September 27, 2023. doi:10.1001/jamanetworkopen.2023.36023

Data
Data available: Yes
Data types: Data (not involving human participants)
How to access data: osf.io/uj56m/
When available: With publication

Supporting Documents
Document types: Statistical/analytic code
How to access documents: osf.io/uj56m/
When available: With publication

Additional Information
Who can access the data: Public
Types of analyses: All analyses
Mechanisms of data availability: Public
